# Diagnosis, treatment and survival from bladder, upper urinary tract, and urethral cancers: real‐world findings from NHS England between 2013 and 2019

**DOI:** 10.1111/bju.15970

**Published:** 2023-02-07

**Authors:** James W.F. Catto, Olena Mandrik, Lewis A. Quayle, Syed A. Hussain, John McGrath, Joanne Cresswell, Alison J. Birtle, Rob J. Jones, Paramananthan Mariappan, Lydia E. Makaroff, Allen Knight, Hugh Mostafid, Jim Chilcott, Peter Sasieni, Marcus Cumberbatch

**Affiliations:** ^1^ Department of Oncology and Metabolism University of Sheffield Sheffield UK; ^2^ Health Economics and Decision Science, School of Health and Related Research University of Sheffield Sheffield UK; ^3^ Department of Urology Sheffield Teaching Hospitals NHS Foundation Trust Sheffield UK; ^4^ Department of Medical Oncology Sheffield Teaching Hospitals NHS Foundation Trust Sheffield UK; ^5^ Department of Urology Royal Devon University Hospitals Foundation Trust, University of Exeter Exeter UK; ^6^ South Tees NHS Hospitals Trust Middlesborough UK; ^7^ Rosemere Cancer Centre Lancashire Teaching Hospitals Preston UK; ^8^ University of Manchester Manchester UK; ^9^ University of Central Lancashire Lancaster UK; ^10^ School of Cancer Sciences, Beatson West of Scotland Cancer Centre University of Glasgow Glasgow UK; ^11^ Department of Urology, Edinburgh Bladder Cancer Surgery Western General Hospital Edinburgh UK; ^12^ Fight Bladder Cancer Oxfordshire UK; ^13^ Patient and Trustee Action Bladder Cancer UK Guildford UK; ^14^ Department of Urology The Royal Surrey County Hospital Guildford UK; ^15^ School of Cancer and Pharmaceutical Sciences King's College London London UK; ^16^ World Bladder Cancer Patient Coalition Brussels Belgium

**Keywords:** bladder cancer, urothelial cell carcinoma, NHS statistics, cancer outcome

## Abstract

**Objective:**

We report NHS England data for patients with bladder cancer (BC), upper tract urothelial cancer (UTUC: renal pelvic and ureteric), and urethral cancers from 2013 to 2019.

**Materials and Methods:**

Hospital episode statistics, waiting times, and cancer registrations were extracted from NHS Digital.

**Results:**

Registrations included 128 823 individuals with BC, 16 018 with UTUC, and 2533 with urethral cancer. In 2019, 150 816 persons were living with a diagnosis of BC, of whom 113 067 (75.0%) were men, 85 117 (56.5%) were aged >75 years, and 95 553 (91.7%) were Caucasian. Incidence rates were stable (32.7–34.3 for BC, 3.9–4.2 for UTUC and 0.6–0.7 for urethral cancer per 100 000 population). Most patients 52 097 (mean [range] 41.3% [40.7–42.0%]) were referred outside the 2‐week‐wait pathway and 15 340 (mean [range] 12.2% [11.7–12.6%]) presented as emergencies. Surgery, radiotherapy, chemotherapy, or multimodal treatment use varied with disease stage, patient factors and Cancer Alliance. Between 27% and 29% (*n* = 6616) of muscle‐invasive BCs did not receive radical treatment. Survival rates reflected stage, grade, location, and tumour histology. Overall survival rates did not improve over time (relative change: 0.97, 95% confidence interval 0.97–0.97) at 2 years in contrast to other cancers.

**Conclusion:**

The diagnostic pathway for BC needs improvement. Increases in survival might be delivered through greater use of radical treatment. NHS Digital data offers a population‐wide picture of this disease but does not allow individual outcomes to be matched with disease or patient features and key parameters can be missing or incomplete.

## Background

Bladder cancers (BCs) and upper tract urinary cancers are common and important public health problems [1]. More than 550 000 new cases are diagnosed yearly worldwide [2]. These tumours are some of the most expensive human cancers to manage [3], and affected individuals report poor experiences [4, 5] with reduced long‐term quality of life [6]. BCs and upper tract urothelial cancers (UTUCs, including ureteric and renal pelvic cancers) often arise following exposure to smoking [7], occupational carcinogens [8], or diesel fumes. In addition, UTUCs may be part of Lynch syndrome or develop after exposure to aristolochic acids [9]. Most BCs, ureteric and renal pelvic cancers, and urethral cancers are urothelial cell carcinoma (UCC) in histological subtype and best stratified into non‐muscle‐invasive (NMI) and muscle‐invasive (MI) cancers. The natural history of NMI and MI cancers differs widely, so treatments reflect risks of progression and metastases [9, 10, 11]. Non‐urothelial bladder and upper tract tumours are often MI with poor outcomes [12].

Population‐wide healthcare data have the potential to offer an objective understanding of the real‐world presentation, treatment, and outcomes. Improvements in connectivity and analytical capacity, combined with lower costs, mean real‐world data are becoming more common and available for interrogation [13]. Proponents suggest real‐world data can replicate prospective randomised trials at low cost and with fewer ethical barriers [14]. As such, they are replacing custom‐built, service‐specific, audit‐focused datasets that are limited in remit and reliant on enthusiasts [15]. However, completion rates may be problematic (missing data), outcomes missing (or misclassified), and patterns of care/outcomes prone to bias. Here, we analyse the most contemporary dataset reflecting BC, UTUC and urethral cancer within NHS England. We hypothesise this will reveal trends in the treatment of these cancers and areas where improvement is needed and possible.

## Methods

### Data Extraction

We extracted data from the National Cancer Registration and Analysis Service (NCRAS) Data Repository, NHS Digital (Appendix S1). We selected cancers, regardless of stage, grade and histology, of the renal pelvis (International Classification of Diseases, 10th edition [ICD‐10] code C65 or D41.1 or [D09.1 with ICD for Oncology, third edition {ICD‐O‐3} code C65], including UCC coded to kidney for all years), ureter (ICD‐10 code C66 or D41.2 or [D09.1 with ICD‐O‐3 code C66]), bladder (ICD‐10 code C67 or D09.0 or D41.4 or [D09.1 with ICD‐O‐3 code C67]) and urethra (ICD‐10 code C68 or [D09.1 with ICD‐O‐3.1 code C68]). As detailed by NCRAS, this excluded urinary tumours of uncertain/unknown behaviour (ICD‐10 code D41.3, D41.7 and D419) as the numbers were small. Cancers were annotated according to the year of first diagnosis, malignant and in situ phenotype, and stage (NMI [pTa, pTis and pT1] or MI [pT2–4]). The route to diagnosis was presented for each cancer and allocated to one of: GP referral, 2‐week‐wait (2ww) pathway (defined as urgent GP referrals with a suspicion of cancer), emergency presentation, outpatients (other), screen detected, inpatient elective (where no earlier information can be found prior to admission from a waiting list, booked or planned), diagnosis by death certificate only, and unknown [16]. Treatments were assigned by NCRAS using Hospital Episode Statistics data, and available for T1–T4 tumours treated from 2013 to 2019. Surgical procedures were allocated using NHS Office of Population Censuses and Surveys Classification of Surgical Operations and Procedures version 4 (OPCS‐4) codes [https://www.datadictionary.nhs.uk/data_elements/opcs‐4_code.html] and ‘Resection’ defined as transurethral resection of bladder tumour (TURBT) for NMI cancer and radical surgery for MI disease. The chemotherapy route was assigned using the Systemic Anti‐Cancer Therapy (SACT) data [https://www.datadictionary.nhs.uk/data_sets/clinical_data_sets/systemic_anti‐cancer_therapy_data_set.html]. Radiotherapy regimen was extracted using the Radiotherapy Data Set (RTDS) [https://www.datadictionary.nhs.uk/data_sets/clinical_data_sets/radiotherapy_data_set.html] and annotated with respect to intent, age, sex, geographic location (Clinical Commissioning Groups), and use of intensity‐modulated radiation therapy.

### Analysis and Statistics

Data were extracted and analysed within Excel for Mac (Version 16.63.1; Microsoft Corp., Redmond, WA, USA). Graphs were generated using Prism 9 for macOS (GraphPad Software Inc., San Diego, CA, USA). Net and Kaplan–Meier computed survivals at 12‐month intervals from diagnosis to 72 months are presented (with CIs). We selected the Kaplan–Meier survival rates and plotted to 5 years after diagnosis, with respect to various tumour features and year of diagnosis. Trends in 2‐ and 3‐year overall survival were plotted for each cancer relative to 2013 outcomes. Survival outcomes were compared using a two‐way anova. Patterns of disease were compared using chi‐squared tests or *t*‐tests, depending upon the variable. All tests were two‐sided, with *P* < 0.05 taken as statistically significant. Statistical comparisons were conducted in RStudio Version 1.6.0 or Prism 9.

### Ethics Approval, Consent and Data Availability

This is publicly available, service‐collected, non‐identifiable data. Ethics Committee approval and consent are not necessary. The full dataset is available to download at: https://www.cancerdata.nhs.uk/getdataout/bladder.

## Results

### New Patients and Incidence

From 2013 to 2019, mean (95% CI) annual case rates were 18 403 (18 266–18 540) for BC, 2288 (2210–2367) for UTUC and 362 (341–383) for new urethral cancer (within 56 286 691 eligible individuals in 2019; Table 1). The annual incidence rates (95% CI) varied between 32.7% (32.2–33.1%) and 34.3% (33.8–34.8%) for BC, 3.9% (3.8–4.1%) and 4.2% (4.1–4.4%) for UTUC, and 0.6% (0.5–0.7%) to 0.7% (0.6–0.8%) for urethral cancer per 100 000 population. Incidence rates appeared stable over time.

**Table 1:** Details of the 146 374 cancers detailed in the NCRAS data repository.

Variable	Bladder, *n* (%)	UTUC, *n* (%)	Urethra, *n* (%)
Total	128 823	16 018	2533
Year			
2013	18 330	2122	331
2014	18 645	2150	352
2015	18 437	2355	321
2016	18 427	2329	390
2017	18 105	2332	367
2018	18 284	2345	377
2019	18 595	2385	395
Histology			
Urothelial	115 166 (89)	15 380 (96)	2533 (100)
Other (non‐urothelial)	4465 (3)	638* (4)	na
Uncertain or unknown	9192 (7)		na
UCC stage			
NMI	83 244 (72)	7280 (47)	2533^†^ (100)
pTa/Tis	57 976 (70)	5265 (72)	
pT1	25 268 (30)	2015 (28)	
MIBC	23 373 (20)	4825 (31)	
Unknown	8549 (7)	3275 (21)	
Other histology stage			
NMI	713 (16)	na	na
MIBC	2944 (66)	na	na
Unknown	808 (18)	na	na
UCC NMI pTa/TIS			
Grade 1	2993 (5)	na	na
Grade 2	27 727 (48)	na	na
Grade 3	8100 (14)	na	na
Unknown	19 156 (33)	na	na

* UTUC non‐urothelial histology not detailed.

^†^ Urethral stage not available.

na, detail not available.

### Prevalence, Sex, Age, Ethnicity and Deprivation

Demographic details were available for BC prevalence only. In 2019, the prevalence of BC was 63 477, 104 192, 142 850 and 150 816 persons at 5, 10, 20 years and at last follow‐up after diagnosis, respectively (Fig. S1). At the last follow‐up, most affected persons were men (*n* = 113 067 [75.0%, rate 406.3/100 000] vs *n* = 37 749 women [25.0%, rate 132.6/100 000]) and aged >75 years (ages: 0–14 years, *n* = 34 [0.02%]; 15–24 years, *n* = 123 [0.1%]; 25–44 years, *n* = 1924 [1.3%]; 45–64 years, *n* = 21 900 [14.5%]; 65–74 years, *n* = 41 718 [27.7%]; 75–84 years, *n* = 55 338 [36.7%]; and >85 years, *n* = 29 779 [19.8%]). The 10‐year prevalence ethnicity data reported 2060 (2.0%) were Asian, 769 (0.7%) Black, 279 (0.3%) mixed race, 95 553 (91.7%) Caucasian, 1019 (1.0%) Other, and 4512 (4.3%) defined as Unknown. There was a roughly equal distribution of cancers with respect to the social deprivation as determined using the Index of Multiple Deprivation (IMD): IMD 1 (most deprived) 23 289 (15.4%) of BCs; 27 027 (17.9%) IMD 2; 31 933 (21.2%) IMD 3; 34 341 (22.8%) IMD 4; and 34 226 (22.7%) for IMD 5 (least deprived).

### Routes to Diagnosis

**Fig. 1:**
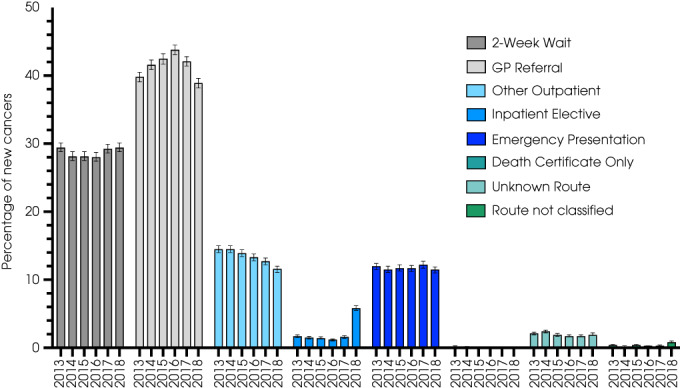
Route to diagnosis for all urinary cancers (including urethral, *n* = 2138; BC, *n* = 110 228; and UTUC, *n* = 13 633) from 2013 to 2018.

Routes to diagnosis were available for patients up to 2018 (*n* = 125 999, Fig. 1). Around one‐quarter (34 630 [27.5%, 95% CI 26.9–28.1%]) were referred via the 2ww pathway (indications for which include visible haematuria or non‐visible haematuria with associated features, such as irritative bladder symptoms or raised leucocytes). A large proportion (52 097 [41.3%, 95% CI, 40.7–42.0%]) were referred by their GP outside the 2ww pathway, whilst 15 340 (12.2%, 95% CI 11.7–12.6%) patients presented as emergencies, usually via Accident and Emergency (between 60% and 67% emergency of cases). Higher staged cancers more commonly presented as emergencies than lower staged cancers (e.g., 18.8% of MIBCs vs 6.4% NMIBCs, and 13.9% of MI UTUCs vs 8.5% of NMI UTUCs, Fig. S2).

There were variations in referral patterns (Table S1) and rates of emergency presentation when mapped by Clinical Commissioning Group/Cancer Alliance. These regional variations were greater for higher staged cancers. For example, for MIBC, the lowest emergency presentation rate was in the Thames Valley (*n* = 3572 [15.1%, 95% CI 14.0–16.4%]) and the highest in North Central and North East London (*n* = 3540 [24.3%, 95% CI 22.9–25.7%]). In 2018, there was a sharp increase in the rate of BCs diagnosed through the inpatient elective route (from 337 [1.6%] to 1178 [5.6%]), suggesting non‐GP referral routes to urological care. This mirrored a decline in GP referral rates (from 8745 [42.0%] to 8175 [38.9%]). Few cancers (107 [<0.2%]) were diagnosed for the first time by death certificate.

### Stage and Grade at Diagnosis

**Fig. 2:**
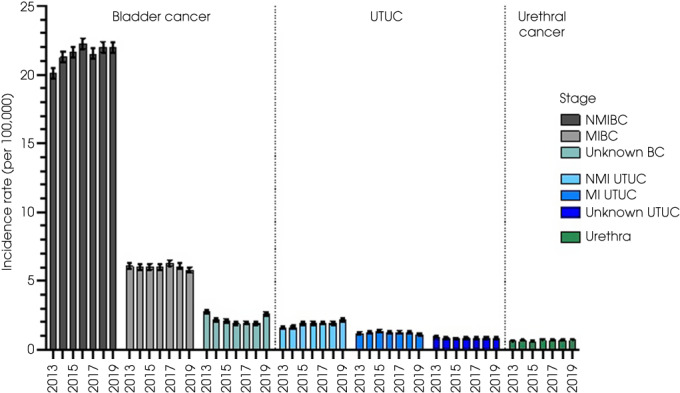
Cancer stage (stratified into NMI, MI, and unknown) at diagnosis for BC, UTUC, and urethral cancer from 2013 to 2019.

The stage at diagnosis was broadly similar over time (incidence rates per 100 000 shown in Fig. 2). Most BCs were NMI (83 957/128 823 [65.2%]) at diagnosis, compared to 26 317 (20.4%) that were MIBC (including 23 373 [20%] UCC MIBC) and 9357 (7.3%) with unknown stage (Table 1). Proportions of NMIBC increased slightly with time (from 67.2% to 70.1%), reflecting better staging data (fewer unknown stage cancers) rather than changes in MIBC rates. Most UTUC were NMI (7280 [45.4%]), compared to 4825 (30.1%) MI and 3275 (20.5%) with unknown stage (Table 1). UTUC had a higher proportion of MI cancer and unknown stage when compared to BC (chi‐squared *P* < 0.001). Tumour grade was only available for pTa/Tis BCs (as most MI and T1 tumours are high‐grade [17]). Most of these BCs were low (Grade 1, 2993 [5%]) or moderate grade (Grade 2, 27 727 [48%]), compared to 8100 (14%) that were high grade (Grade 3). Grade was missing in 19 156 (33%). Urethral cancers were not sub‐staged.

### Treatment

**Fig. 3:**
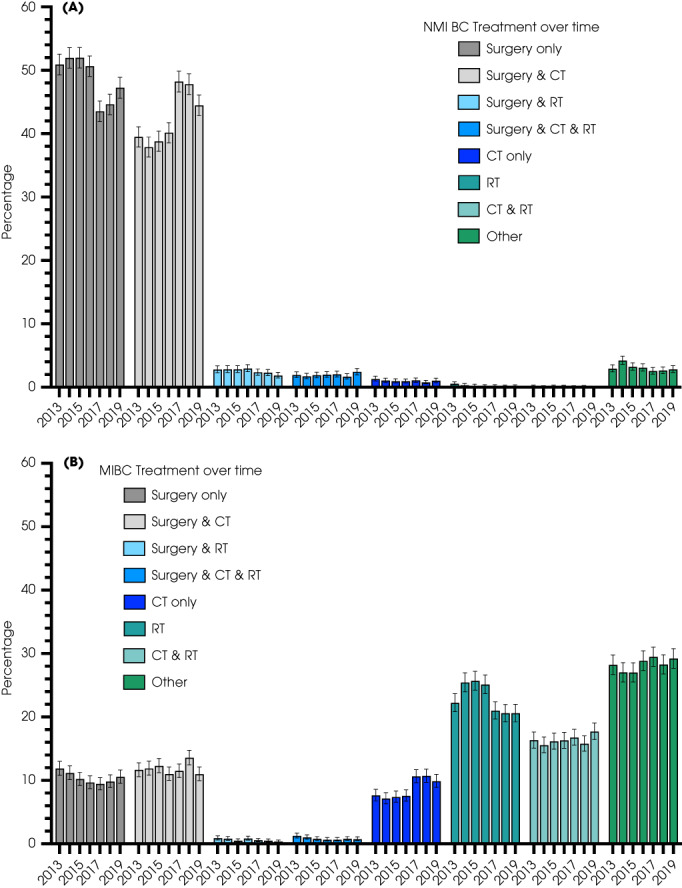
Distribution of individual treatment(s) for (**A**) 25 268 pT1 NMIBCs and (**B**) 23 373 MIBCs from 2013 to 2018. CT, chemotherapy; RT, radiotherapy.

**Fig. 4:**
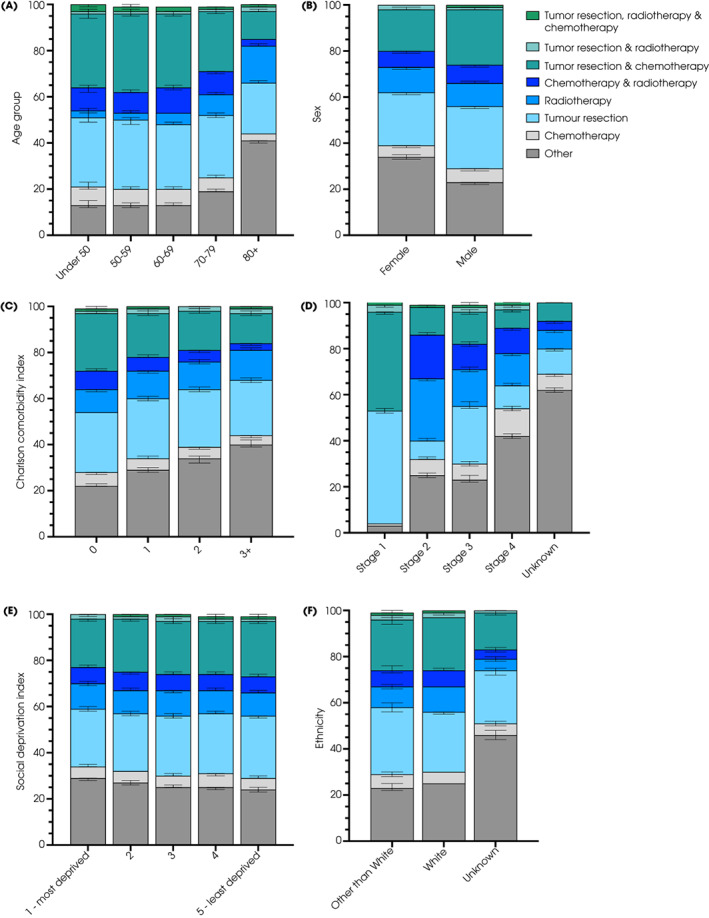
Use of chemotherapy, radiotherapy, surgery in isolation or combination in patients with BC from 2013 to 2018. Percentage of individuals receiving each option is shown according to (**A**) age, (**B**) sex, (**C**) Charlson Comorbidity Index, (**D**) stage, (**E**) social deprivation, and (**F**) ethnicity.

Treatments received in isolation or combination were detailed according to disease stage, patient factors and Cancer Alliance for BC. Distributions varied with stage (Fig. 3 and Table S2), over time, patient age, comorbidity and social deprivation (Fig. 4) and between Cancer Alliances (Figs. S3–S5).

#### Non‐Muscle‐Invasive BC Treatments

Treatment choices were available for all 25 268 (100%) patients with pT1 urothelial cancers between 2013 and 2019. Most patients (Fig. 3A) received surgery (including TURBT) alone (12 307 [range 43.5–52.0%]) or surgery with chemotherapy (10 710 [range 37.9–48.2%]). Trends showed that the latter was replacing surgery alone. A few patients received chemotherapy only (255 [range 0.7–1.3%]) [18] and radiotherapy (either alone or in combination). Information about type of chemotherapy agent (and/ or use of BCG immunotherapy) was not available in this dataset.

#### Non‐Muscle‐Invasive UTUC Treatment

Treatment choices were available for 2015 patients with NMI UTUC from 2013 to 2019. Most patients received surgery alone (1288 [range 57.1–66.8%]) or in combination with chemotherapy (159 [range 5.4–9.9%]). The use of combined surgery and chemotherapy rose from 15 (5.4%) in 2013 to 29 (9.9%) in 2019, perhaps reflecting completed trials [19].

#### Muscle‐Invasive BC Treatments

Treatment choices were recorded for 23 373 patients with urothelial MIBC from 2013 to 2019. Chemotherapy was used in 8830 (37.8%), radiotherapy in 9525 (40.8%) and radical surgery in 5533 (23.7%) patients (Fig. 3B and Fig. S3). The use of chemotherapy increased from 36.9% in 2013 to 40.8% in 2018. For patients receiving radiotherapy, 5358 (56.3%) received this alone, 3826 (40.2%) with chemotherapy, 145 (1.5%) with surgery, and 196 (2.1%) with both chemotherapy and surgery. Radiotherapy intent was equally split between radical, palliative and other regimens, mostly did not vary by sex or age, and there was an apparent move to intensity‐modulated radiation therapy (Fig. S5) and there were differences between Cancer Alliances. For those who underwent radical surgery, 2424 (43.8%) had surgery alone, 2768 (50.0%) also received chemotherapy, 145 (2.6%) also received radiotherapy, and 196 (3.5%) had all three treatments. Chemotherapy was the only treatment in 1708 (7.3%) patients. Between 27% and 29% (*n* = 6616) of patients received ‘Other’ treatment, likely to reflect palliative non‐radical options [20].

#### Muscle‐Invasive UTUC Treatments

Treatment choices were recorded for 4825 patients with MI UTUC from 2013 to 2019. Surgery was used in 3781 (78.4%), chemotherapy in 1435 (29.7%), and radiotherapy in 402 (8.3%) patients. The use of chemotherapy with surgery doubled from 99 (15.4%) in 2013 to 202 (30.7%) in 2018 [21]. For patients receiving surgery, 2479 (53.3%) had surgery alone, 1045 (20.0%) also received chemotherapy, 132 (2.8%) also received radiotherapy, and 125 (2.6%) had all three treatments. Radiotherapy was used in isolation in 72 (1.4%), in combination with chemotherapy in 73 (1.5%), and in combination with surgery and chemotherapy in 125 (1.5%). A few patients (192, 3.7%) received only chemotherapy.

#### Urethral Cancer Treatment

No treatments for urethral cancer were recorded.

### Survival Outcomes

**Fig. 5:**
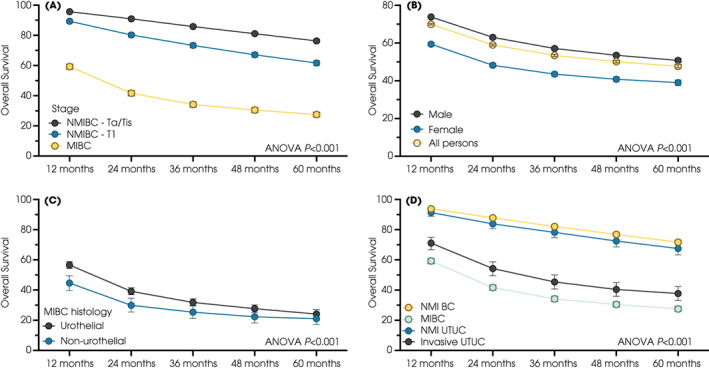
Overall survival plotted using the Kaplan–Meier method for patients with (**A**) BC with respect to stage, (**B**) sex, (**C**) MIBC with respect to histology, and (**D**) UCC with respect to location (bladder [BC] and upper urothelial tract [UTUC]) and stage.

Overall survival was determined for 98 031 patients with BC and 16 018 with UTUC, treated from 2013 to 2018. Comparisons concerning the year of diagnosis showed no change in survival rates, whether for all BCs, only MIBCs or UTUC (Fig. S6). For BC, overall survival differed markedly regarding the presence of muscle invasion (anova *P* < 0.001, pTa/Tis vs pT1 vs MIBC, Fig. 5A). For NMI, survival rates were similar for Grade 1 and 2 cancer (anova *P* = 0.9) and worse for Grade 3 cancers (anova *P* = 0.002) at 60 months. Women had worse outcomes than men when all stages were combined (Fig. 5B, anova *P* < 0.001). For MIBC, non‐urothelial tumours had lower short‐term survival rates than urothelial cancer, although outcomes at 5 years were broadly similar (24.1% [95% CI 21.4–27.0%] for UCC vs 21.0% [17.1–25.3%] for non‐UCC, anova *P* < 0.001, Fig. 5C). Patients with MIBC had lower survival rates than those with MI UTUC (anova *P* < 0.001). Comparisons with other common cancers revealed that MIBC had worse survival outcomes than many and that NMIBC had a similar survival to all prostate cancers (Fig. S7). Trend analysis highlighted a lack of improvement for BC. For example, the relative change in overall survival rates was 0.97 percentage points (95% CI 0.97–0.97) and 0.99 (95% CI 0.99–0.99) at 3 years, Fig. S8 for BC, in contrast to other cancers (e.g., increased survival from oesophageal cancer [relative 1.06 percentage points, 95% CI 1.06–1.06] at 2 and 3 years).

## Discussion

The NCRAS data repository provides important information on incidence, prevalence, routes to diagnosis, staging and treatment for BC, UTUC and urethral cancers. The repository includes NMI disease (which had previously been excluded), and improvements have reduced the amount of missing data (e.g., stage missing in only 7% for BC and 21% for UTUC, compared to >30% previously [22, 23, 24]). This more complete dataset delivers a different picture of BC from that reported before. For example, there is a much higher incidence of BC (average annual case rate was >18 000 in 2013–2019 compared to 10 000 previously [22]), >150 000 persons are living with a prior diagnosis of BC (Fig. S1), around one‐third of NMI patients have lamina propria invasion (pT1). There are wide variations between different Cancer Alliances.

The route to diagnosis findings question current NHS pathways. In particular, most BCs and UTUCs were not diagnosed via 2ww referrals (in contrast to other common cancers, e.g., 48% for head and neck and 48% for prostate cancers were referred as 2ww priorities, Fig. S9). More BC/UTUCs were referred from their GP as non‐2ww cases (41.3%), so it will be essential to understand which symptoms were responsible for this. Haematuria outcome studies report low rates of BC in persons with non‐visible haematuria. That risk varies dramatically between different populations with similar symptoms (e.g., men vs women, smokers vs non‐smokers) [25]. Patient surveys reveal affected individuals often complain of irritative bladder symptoms (dysuria, frequency) rather than or prior to developing haematuria [26]. Changes in routine blood tests can be seen 6–9 months before a diagnosis of BC and kidney cancer [27]. The 2ww‐referral criteria should be adjusted to include patient features (sex, smoking history, other risk factors) and symptoms (irritative LUTS), as NHS resources and attention are focused on these pathways. BCs diagnosed via the 2ww route have better outcomes (70% overall survival at 36 months) than those via routine GP referrals (60% at 36 months) [https://www.cancerdata.nhs.uk/routestodiagnosis/survival], and patients with haematuria face fewer delays than those with other symptoms [28]. Another key observation is that around one in 10 patients were diagnosed at an emergency admission. Presumably, these patients had undiagnosed symptomatic or non‐symptomatic cancers in the community, and this emergency represents a failure to find these cancers at this earlier stage. Consequently, there is a direct relationship between rising stage and rates of emergency diagnosis, and wide variations are seen between Clinical Commissioning Groups (Fig. S2C). It will be important to learn from Clinical Commissioning Groups that perform better than others and undertake quality improvement initiatives [29]. Reassuringly, few patients were diagnosed through their death certification (Fig. S10), suggesting a high symptomatic presentation rate and a lack of latent disease.

In general, treatment patterns reflected National Institute for Health and Care Excellence (NICE) and European Association of Urology (EAU) guidance [11, 30]. For example, for NMIBC, there appeared to have been a switch to include chemotherapy with TURBT [31]; for MIBC the rates of radiotherapy‐only were falling (perhaps with broader use of chemoradiotherapy [32]) and more participants undergoing radical cystectomy also received chemotherapy [11]. These findings are similar to those from Cancer Research UK for 2013–2014 for radical cystectomy (23.3% vs weighted average 22.7% for Stage 2–4 BC) and higher than those for radiotherapy (40.1% vs 28.15% and 36.6% vs 28.4% for Stage 2–4 BC, respectively) [https://www.cancerresearchuk.org/health‐professional/cancer‐statistics/statistics‐by‐cancer‐type/bladder‐cancer/diagnosis‐and‐treatment#heading‐One]. That many patients with MIBC did not receive radical treatment is disappointing and matches a more detailed report for non‐metastatic MIBC [20]. Whilst associating treatments with patient factors for MIBC is not possible, the population data suggest radical treatment was less likely to be used with increasing age and comorbidity, with unknown ethnicity, with female sex and with higher social deprivation (Fig. 4). There were marked differences in treatment patters between Cancer Alliances, and (as reported previously) women had worse survival outcomes than men [33].

Various limitations require discussion. First, whilst population‐wide data deliver large sample sizes for comparisons, they are limited in terms of granularity. Individual patient factors are unknown, observations need further work to understand (e.g., the reason for referral outside the 2ww), and there can be limited levels of detail or granularity (e.g., differences in NIMBC/MIBC rates between regions). This is demonstrated in the treatment data, for which some details are unclear, and there are no data for pTa/pTis cancers. Radiotherapy allocations are often unknown/other [34] and there is no record of immunotherapy. This is particularly impactful in the analysis of NMIBC, in which intravesical BCG is an important treatment. BCG is often not recorded on the hospital Trusts’ e‐prescribing system because a urologist, not an oncologist, administers it. The collection of BCG treatment is out of scope in SACT, version 3.0.

Importantly it is not possible to match treatments and patient factors with outcomes to understand the relationships between these factors. Second, despite added work, data remain missing, and areas of high missingness may be those where work is needed most urgently (either in treatment pathways or geographic providers). Regardless of these limitations, population‐wide data offer a powerful resource superior to self‐submitted data [15]. Whilst the latter were useful for comparative outcomes and the design of clinical trials [35, 36], population‐wide data allow comparisons between diseases and offer the power to identify problems with disease pathways.

A more complete picture of BC and UTUC offers insights into solutions that could improve outcomes. The higher than previously reported incidence suggests early detection programmes could be possible and effective/cost‐effective if implemented among populations with a high rate of undiagnosed disease. This is clearly present in some populations of BC [37, 38, 39]. At the same time, the high prevalence of low‐grade NMIBC (which has a low risk of BC death [40]) highlights the importance of estimating the benefit–harm ratio for BC screening programmes before their implementation without overwhelming diagnostic services. Detecting disease early may decrease the load on both the emergency and the 2ww pathways (which require substantially more NHS resources [41]) and lower late‐stage presentation, health system resource use, and costs.

## Conclusions

The NCRAS dataset offers key insights into contemporary BC, UTUC and urethral cancer within England. Whilst the dataset does not allow matching for individual outcomes or patient features and key parameters can be missing or incomplete, the findings of this study allow us to question the referral pathways, highlighting that one in 10 patients present as emergencies, which suggests that the diagnostic pathway (and 2ww criteria) for BC needs reviewing. Improvements in survival might be delivered through greater use of radical treatment.

## Author Contributions

Drs Catto, Mandrik, Quayle and Makaroff had full access to all of the data in the study. Drs Catto and Mandrik take responsibility for the integrity of the data and the accuracy of the data analysis. *Concept and design*: Catto, Mandrik, Quayle and Makaroff. *Acquisition, analysis, or interpretation of data*: Catto, Mandrik, Quayle, Hussain, McGrath, Cresswell, Birtle, Jones, Knight, Mostafid, Sasieni, and Cumberbatch. *Drafting of the manuscript*: Catto, Mandrik, and Quayle drafted the initial manuscript. All authors then read, edited, and commented on the manuscript. *Intellectual content*: Catto, Mandrik and Makaroff. *Obtained funding*: Catto, Sasieni and Cumberbatch. *Administrative, technical, or material support*: Mandrik, Quayle and Makaroff.

## Funding

This study was supported by the Yorkshire Cancer Research UK (Grant number RA/2019/R1/004). James W.F. Catto is funded by a UK National Institute for Health Research (NIHR) Research Professorship.

## Disclosures of Interest

James Catto reported receiving reimbursement for consultancy from AstraZeneca, Ferring, Roche, and Janssen; speaker fees from Bristol Myers Squibb, Merck Sharp & Dohme, Janssen, Astellas, Nucleix, and Roche; honoraria for membership in advisory boards from Ferring, Roche, Gilead, Photocure, Bristol Myers Squibb, QED Therapeutics, and Janssen; and research funding from Roche. Syed Hussain has received reimbursement for consultancy from Pierre Fabre, Bayer, Janssen Oncology, Roche, Merck, Bristol‐Myers Squibb, AstraZeneca, Pfizer, Astellas, GSK; research funding from CRUK, MRC/NIHR, Boehringer Ingelheim, Roche, Janssen‐Cilag, Pierre Fabre; support for attending meetings and/or travel from Janssen‐Cilag, Bayer, Boehringer Ingelheim, Pierre Fabre, Pfizer, Roche, Bristol‐Myers Squibb, AstraZeneca, MSD Oncology. John McGrath has received educational funding from Intuitive Surgical. Robert Jones reported receiving reimbursement for consultancy from MSD, Merck Serono, Pfizer, Roche, Astellas, Clovis, Exelixis, Ipsen, BMS, Bayer, AstraZeneca; speaker fees from MSD, Merck Serono, Pfizer, Roche, Astellas, BMS, Ipsen, AstraZeneca; research funding from Exelixis, Astellas, Pfizer, AstraZeneca, Roche, GSK. Alison Birtle has received speaker fees from Roche, Sanofi Genzyme, MSD, Janssen, research funding from Sanofi Genzyme with advisory board participation with AstraZeneca, Beyer, Janssen and Roche. Lydia Makaroff is an employee of Fight Bladder Cancer, which has received financial support from Astellas, AstraZeneca, BMS, Ferring, Janssen, Medtec, Merck, MSD, Pfizer, Prokarium, Roche, Sanofi, and SeaGen.

**Abbreviations**
2ww: 2‐week wait
BC: bladder cancer
ICD‐10: 10th edition
ICD‐O‐3: International Classification of Diseases for Oncology, third edition
IMD: Index of Multiple Deprivation
MI: muscle invasive
NCRAS: National Cancer Registration and Analysis Service
NMI: non‐muscle invasive
SACT: systemic anti‐cancer therapy
TURBT: transurethral resection of bladder tumour
UCC: urothelial cell carcinoma
UTUC: upper tract urothelial cancer

## Supporting information


**Appendix S1.** Dictionary of source data used in this report.
**Fig. S1.** Prevalence of BC and UTUC 10‐years since diagnosis.
**Fig. S2.** Emergency presentation of BC.
**Fig. S3.** An overview of the use of chemotherapy, radiotherapy, and radical surgery in patients with invasive BC from 2013 to 2018.
**Fig. S4.** Use of chemotherapy, radiotherapy, surgery in isolation and combination in patients with BC from 2013 to 2018 according to cancer alliance.
**Fig. S5.** Patterns of radiotherapy use for advanced BC.
**Fig. S6.** Overall survival for (**A**) all urothelial BCs, (**B**) urothelial MIBCs, and (**C**) all UTUCs according to the year of diagnosis from 2013 to 2018.
**Fig. S7.** Average survival rates from 2013 to 2018 for (**A**) common cancers (in **B**) BC and UTUC are sub‐stratified by stage within the National Cancer Registration and Analysis Service Data Repository.
**Fig. S8.** Trends in overall survival for multiple cancers since 2013.
**Fig. S9.** Routes to diagnosis for (**A**) BCs and UTUCs, (**B**) kidney (renal) cancer, (**C**) head and neck cancer, and (**D**) prostate cancer.
**Fig. S10.** Rates of certification through death certification diagnosis.
**Table S1.** Routes to diagnosis for NMIBC and MIBC for each cancer alliance (from https://www.cancerdata.nhs.uk/routestodiagnosis/index).
**Table S2.** Treatment choices for all cancers within this dataset.
