# Supplementary material for: Diagnosis, treatment and survival from bladder, upper urinary tract, and urethral cancers: real‐world findings from NHS England between 2013 and 2019
Source: BJU Int. 2023 Feb 7;131(6):734–44. doi: 10.1111/bju.15970 (PMC10952282; doi:10.1111/bju.15970)
Supplement: Supplementary file 1 — Appendix S1. Dictionary of source data used in this report. Fig. S1. Prevalence of BC and UTUC 10‐years since diagnosis. Fig. S2. Emergency presentation of BC. Fig. S3. An overview of the use of chemotherapy, radiotherapy, and radical surgery in patients with invasive BC from 2013 to 2018. Fig. S4. Use of chemotherapy, radiotherapy, surgery in isolation and combination in patients with BC from 2013 to 2018 according to cancer alliance. Fig. S5. Patterns of radiotherapy use for advanced BC. Fig. S6. Overall survival for (A) all urothelial BCs, (B) urothelial MIBCs, and (C) all UTUCs according to the year of diagnosis from 2013 to 2018. Fig. S7. Average survival rates from 2013 to 2018 for (A) common cancers (in B) BC and UTUC are sub‐stratified by stage within the National Cancer Registration and Analysis Service Data Repository. Fig. S8. Trends in overall survival for multiple cancers since 2013. Fig. S9. Routes to diagnosis for (A) BCs and UTUCs, (B) kidney (renal) cancer, (C) head and neck cancer, and (D) prostate cancer. Fig. S10. Rates of certification through death certification diagnosis. Table S1. Routes to diagnosis for NMIBC and MIBC for each cancer alliance (from https://www.cancerdata.nhs.uk/routestodiagnosis/index). Table S2. Treatment choices for all cancers within this dataset. [file BJU-131-734-s001.pdf]

Supplementary Material

Diagnosis, treatment and survival from Bladder, Upper Urinary Tract and Urethral Cancers: Real world findings from NHS England between 2013 and 2019

SUPPLEMENTARY CONTENT

1. Appendix 1. Dictionary of Source data used in this report
2. Supplementary figures 1 to 10
3. Supplementary tables 1 to 2.

Appendix 1. Data sources used in this report

GDO UCC data (2013-19)
https://www.cancerdata.nhs.uk/getdataout/bladder

- Bladder, UTUC and Urethral cancer: Case numbers and incidence rates 2013-2019
- Split into Malignant and in situ vs. uncertain
- Stage: NMI, MIBC, unknown, all stages. NMI split into Ta, Tis, Ta/Tis, T1 and NOS
- Grade: all, grade 1, grade 2, grade 3, unknown
- Route of diagnosis (2013-18)
- Survival: Net and Kaplan-Meier survival up to 84 months

GDO All 15 cancers data
https://www.cancerdata.nhs.uk/getdataout/data

Prevalence
https://www.cancerdata.nhs.uk/prevalence

- Prevalence in 2019 for BC only
- vs. age, ethnicity, sex, stage (invasive vs/ invasive/non-invasive), CCG/Cancer Alliance, IMD, Years since diagnosis

Routes to diagnosis details (2006-16)
https://www.cancerdata.nhs.uk/routestodiagnosis

- vs. Age, IMD, Alliance, Stage (all vs. in situ BC)

Survival
https://www.cancerdata.nhs.uk/survival/indexofcancersurvival

- Survival by CCG
- Trends in survival

https://digital.nhs.uk/data-and-information/publications/statistical/cancer-survival-in-england/cancers-diagnosed-2015-to-2019-followed-up-to-2020

- Survival by sex, stage, age
- Survival for other cancers

Treatment (2013-19)
https://www.cancerdata.nhs.uk/treatments

- Treatments as individual options
- Treatment combinations
- Vs. Age, Ethnicity, CCI, IMD, Gender, Stage and Year of diagnosis

Radiotherapy used
https://www.cancerdata.nhs.uk/radiotherapy/dashboard

- Vs. Cancer alliance, disease group. Age group, intent, year, sex

Emergency presentation
https://www.cancerdata.nhs.uk/emergencypresentations

Staging data
https://www.cancerdata.nhs.uk/stage_at_diagnosis

- Geography (alliance, CCG etc.)
- Trends (%) per year
- Trends by site

Supplementary figures

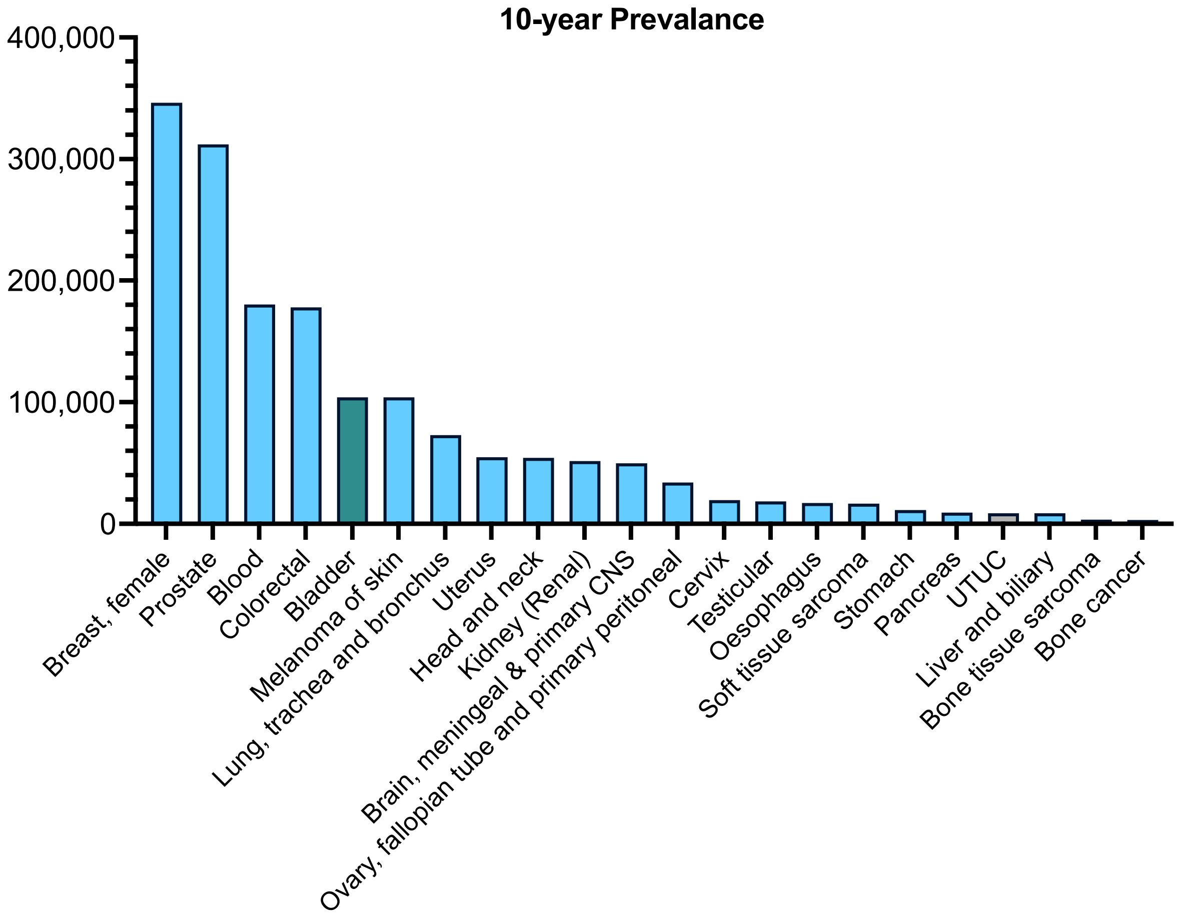

Supplementary figure 1. Prevalence of Bladder Cancer and UTUC 10-years since diagnosis. In 2019, there were 104,192 persons living with bladder cancer (green) and 8,625 living with UTUC (grey) 10 years after diagnosis.

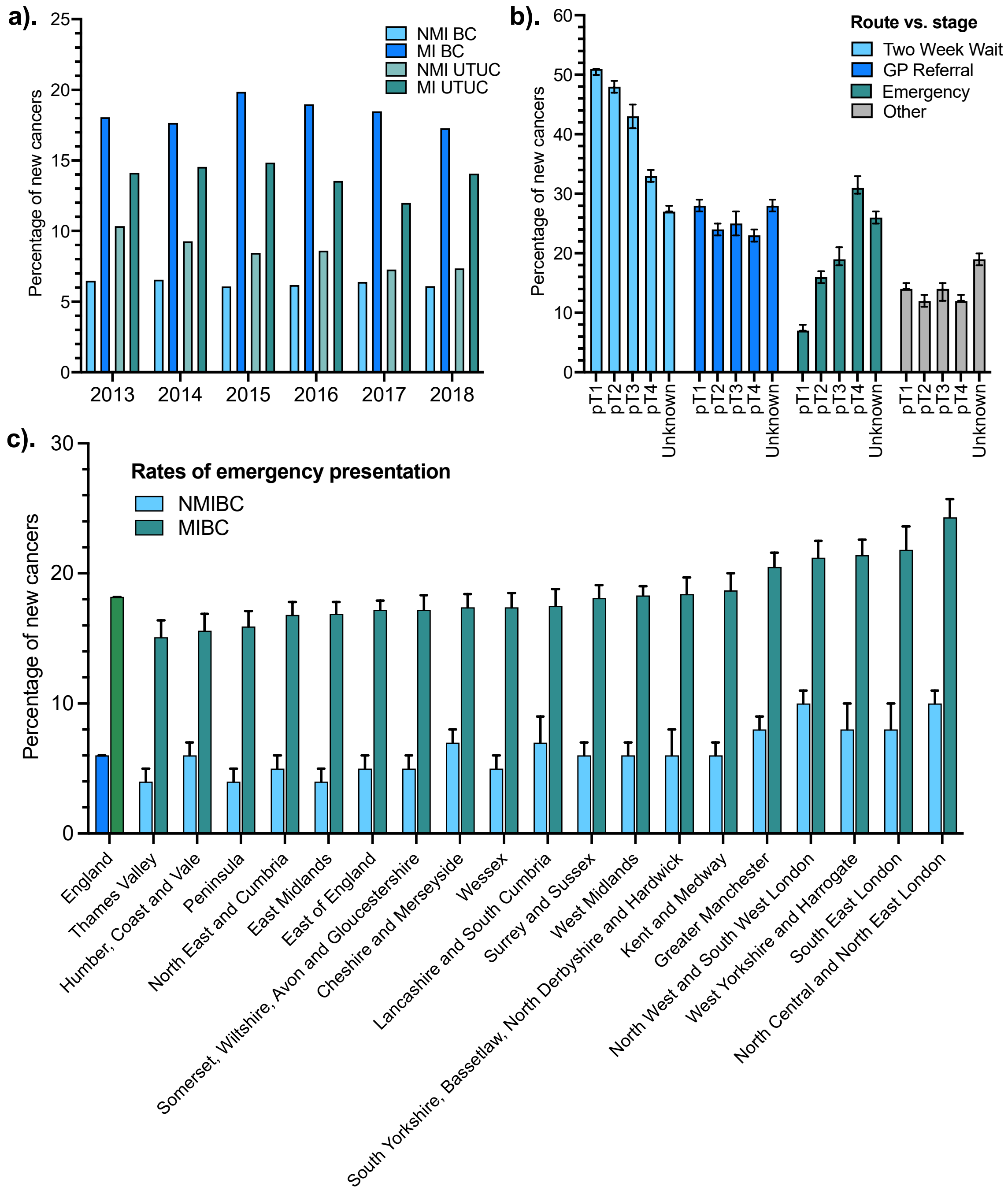

Supplementary figure 2. Emergency presentation of bladder cancer. a). Percentage of non-muscle invasive (NMI) and muscle invasive (MI) bladder cancers (BCs) and UTUCs presenting as an emergency from 2013 to 2018, b). Route to diagnosis for bladder cancer with respect to tumour stage (tumour from 2013-18). c). Percentage of MI and NMI bladder cancers presenting as an emergency for each NHS cancer alliance (compared to average for England in bold).

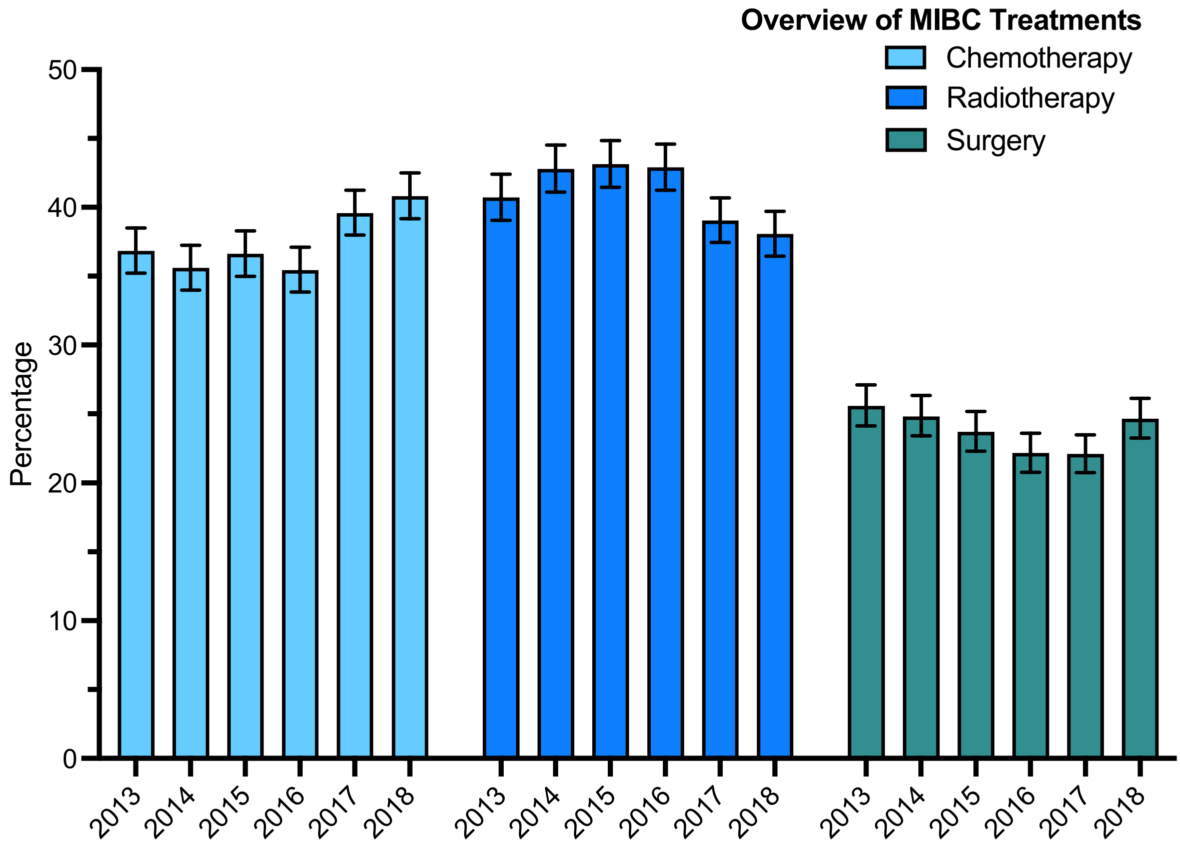

Supplementary figure 3. An overview of the use of chemotherapy, radiotherapy and radical surgery in patients with invasive bladder cancer from 2013 to 2018. Percentage of patients receiving one or more of each treatment shown.

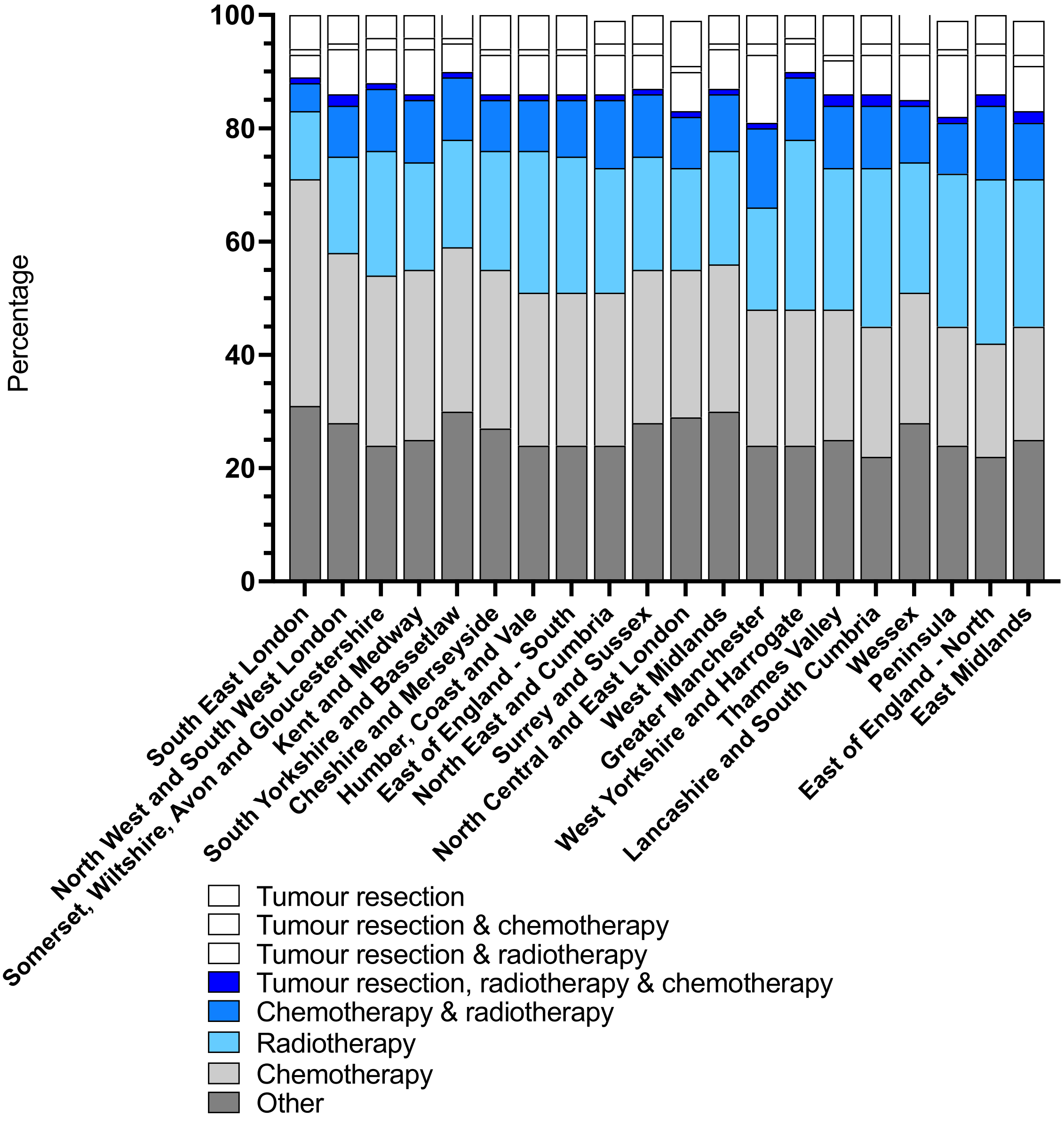

Supplementary figure 4. Use of chemotherapy, radiotherapy, surgery in isolation and combination in patients with bladder cancer from 2013 to 2018 according to cancer alliance.

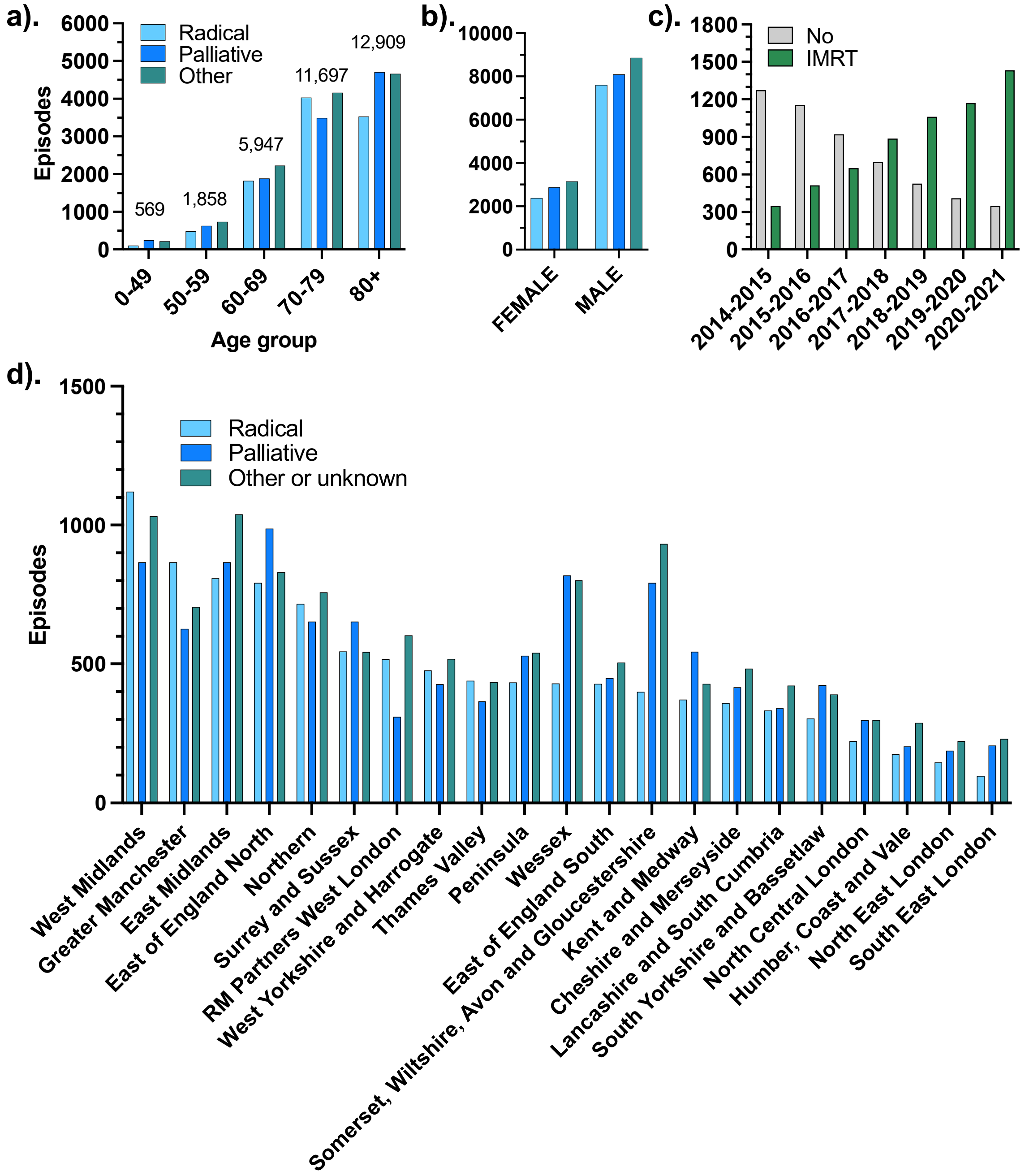

Supplementary figure 5. Patterns of radiotherapy use for advanced Bladder cancer. Radiotherapy regimens for patients with BC from 2009 to 2021 stratified by a). patient age and b). sex, c). The proportion of treatments using intensity modulated radiotherapy for years from 2014 to 2021, d). Use of radical, palliative and other radiotherapy regimens from 2009-2021 according to each NHS cancer alliance.

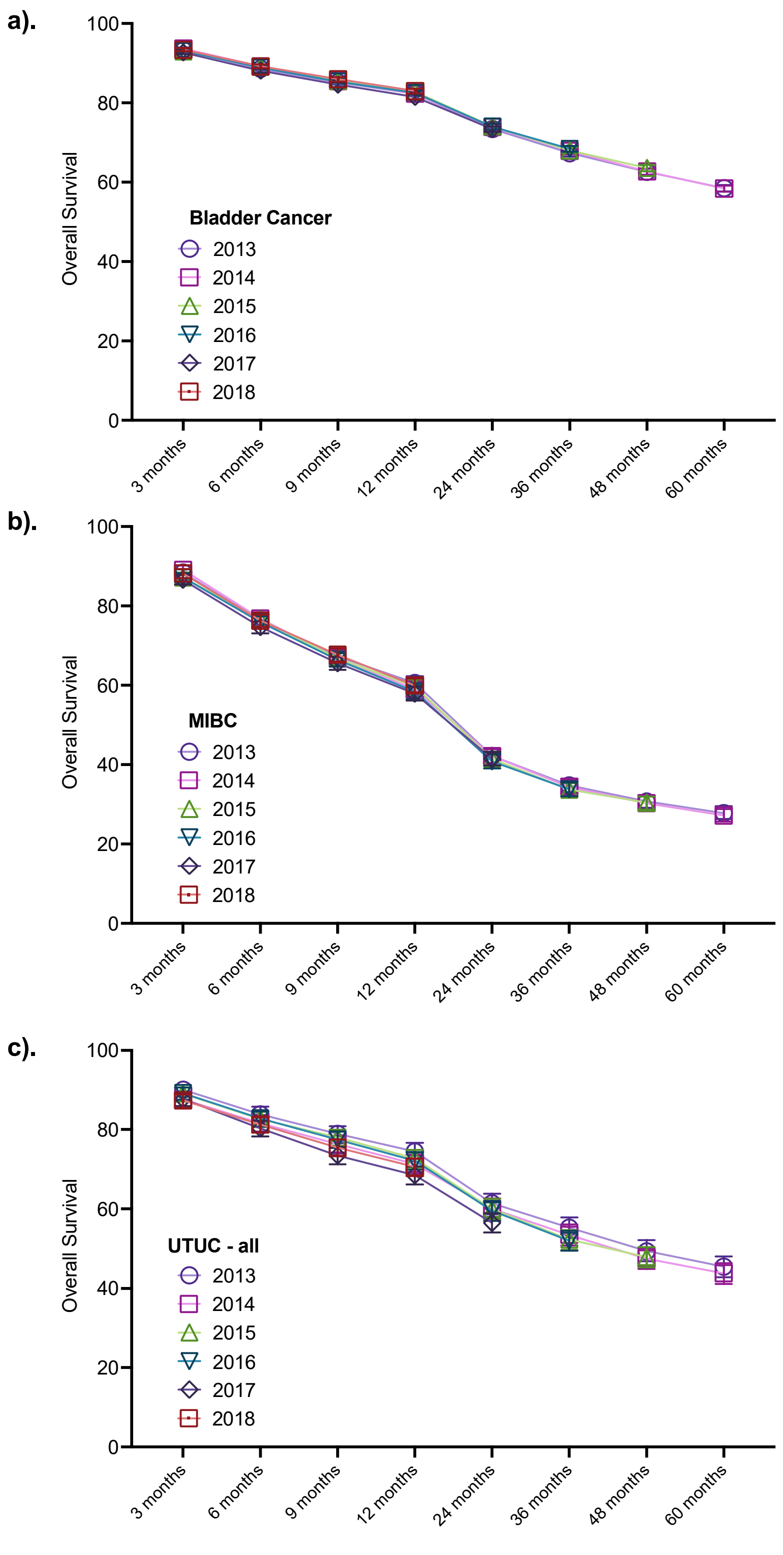

Supplementary figure 6. Overall survival for a). all urothelial bladder cancers, b). urothelial muscle invasive bladder cancers, and c). all upper tract urothelial cancers according to the year of diagnosis from 2013 to 2018.

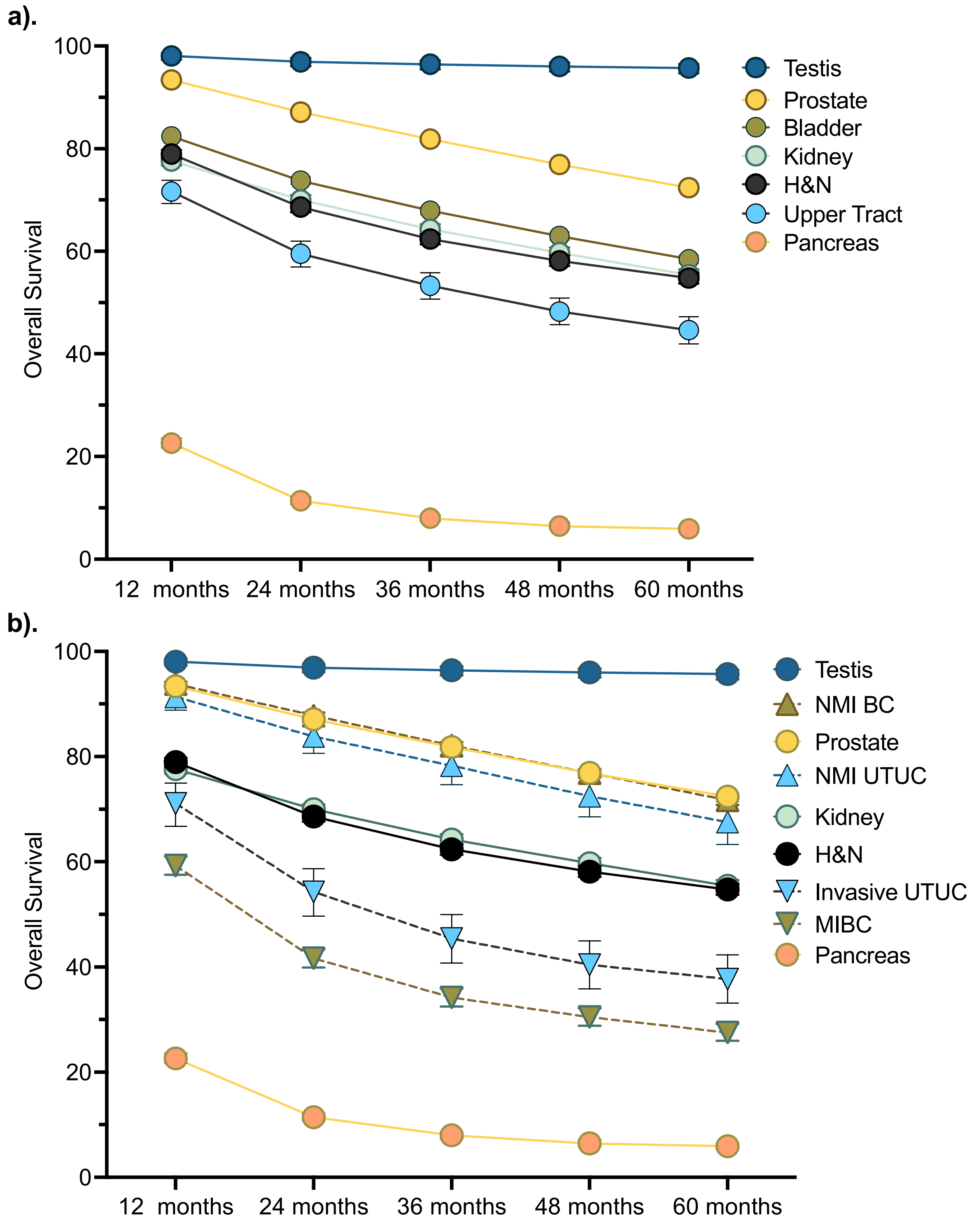

Supplementary figure 7. Average survival rates from 2013 to 2018 for a). common cancers (in b). BC and UTUC are sub-stratified by stage) within the National Cancer Registration and Analysis Service Data Repository.

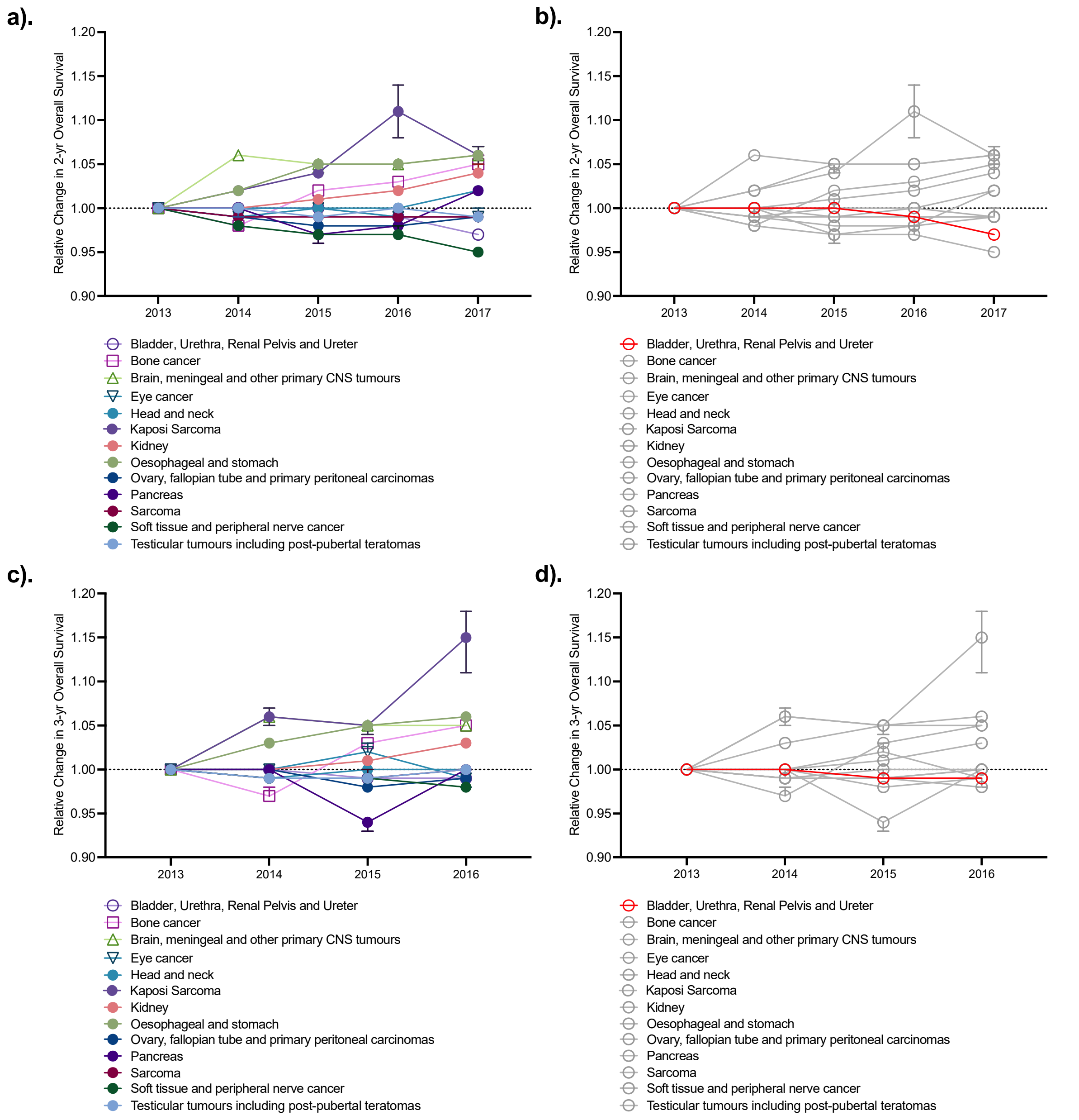

Supplementary figure 8. Trends in overall survival for multiple cancers since 2013. For each cancer, the relative survival at 2-years (a. and b.) and 3-years (c. and d.) after diagnosis are shown for the years 2013 to 2018. For illustrative purposes, graphs are duplicated with all cancers coloured (a. and c.) or highlight to show bladder cancer (b. and d.).

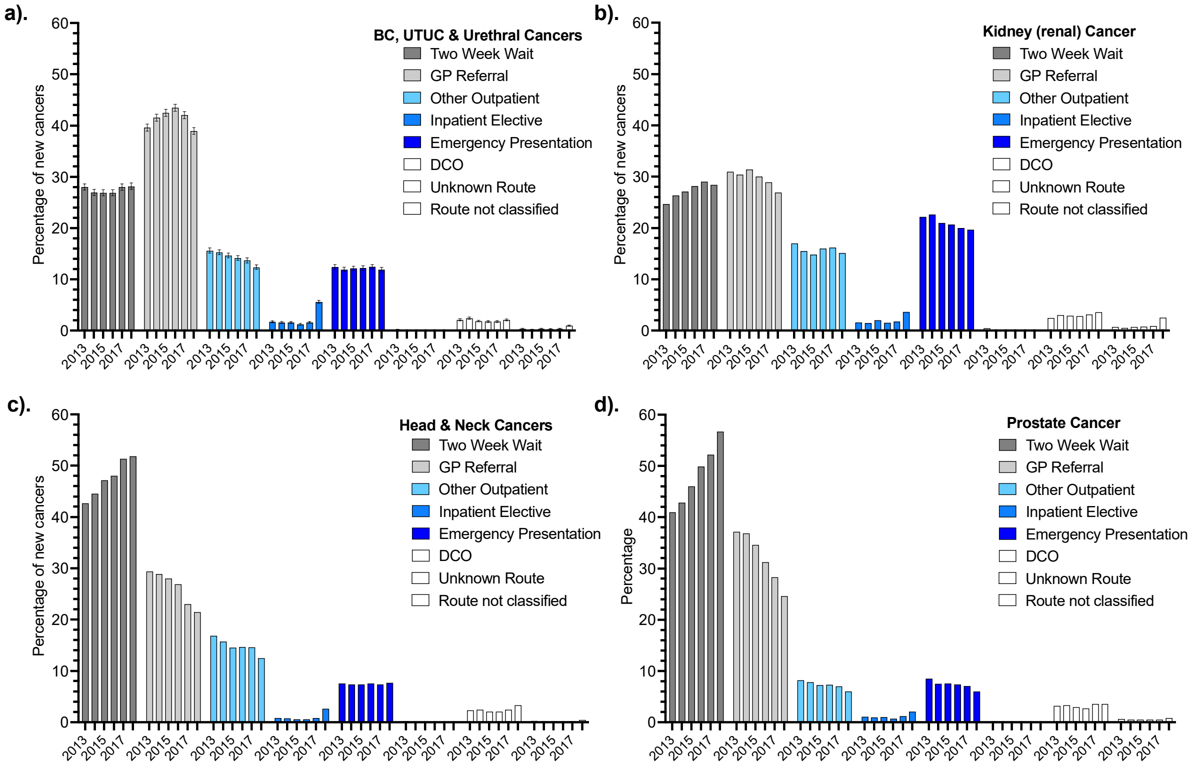

Supplementary figure 9. Routes to diagnosis for a). bladder and UTUC cancers, b). kidney (renal) cancer, c). head and neck cancer and d). prostate cancer.

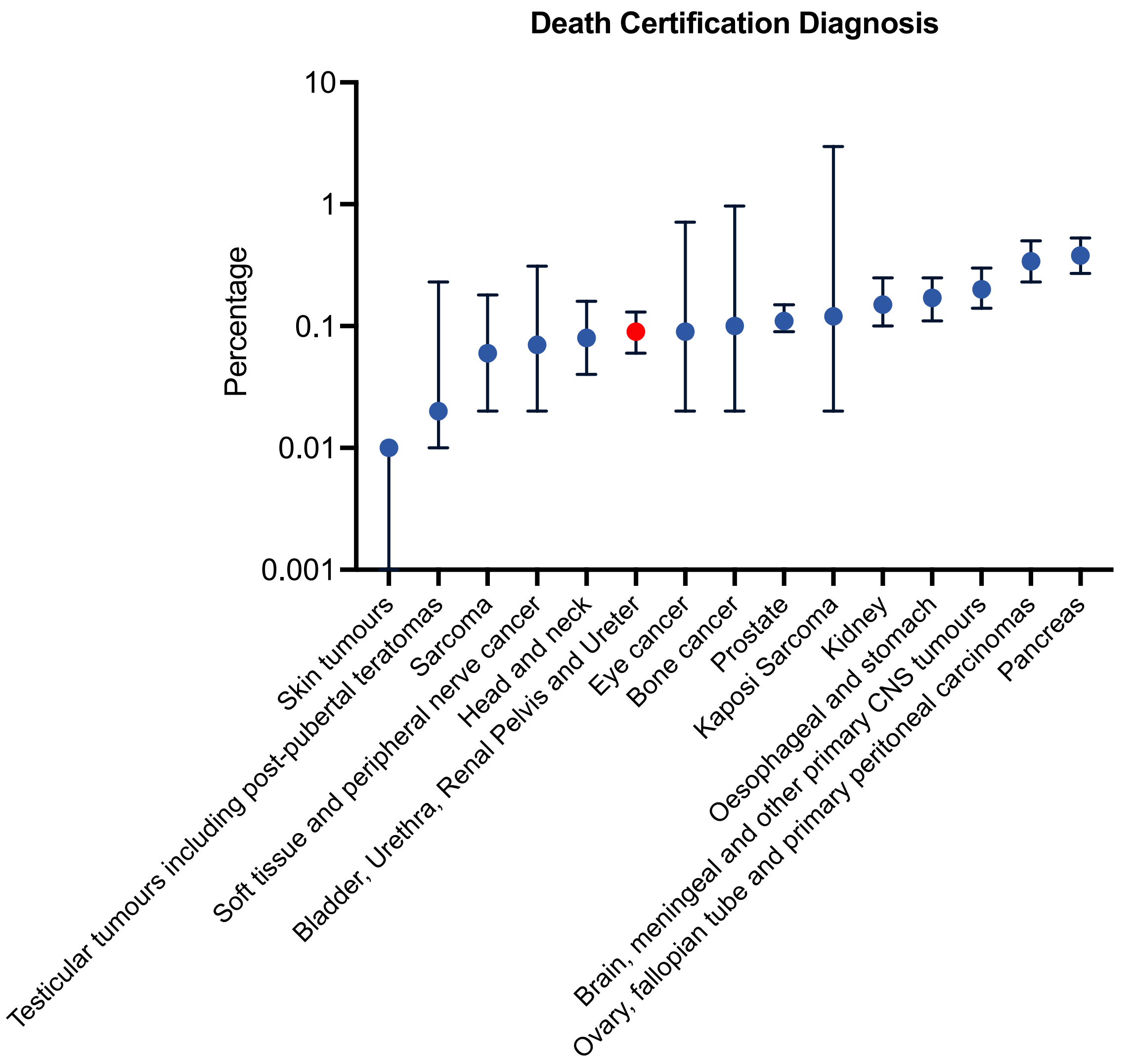

Supplementary figure 10. Rates of certification through death certification diagnosis.

NMI BC	Number of NMI BC cases	Two Week Wait	95% CI		GP referral	95% CI		Other Outpatient	95% CI		Inpatient Elective	95% CI		Emergency presentation	95% CI		Death Certificate Only	95% CI		Unknown	95% CI	
Cheshire and Merseyside	**1,918**	***18%***	*17%*	*20%*	***52%***	*50%*	*54%*	***21%***	*19%*	*23%*	***1%***	*1%*	*2%*	***7%***	*6%*	*8%*				***1%***	*1%*	*2%*
East Midlands	**3,583**	***24%***	*23%*	*25%*	***48%***	*47%*	*50%*	***20%***	*19%*	*22%*	***1%***	*1%*	*2%*	***4%***	*4%*	*5%*				***2%***	*1%*	*2%*
East of England	**5,563**	***25%***	*24%*	*26%*	***51%***	*50%*	*53%*	***12%***	*12%*	*13%*	***3%***	*3%*	*4%*	***5%***	*5%*	*6%*				***3%***	*3%*	*4%*
Greater Manchester	**2,895**	***16%***	*14%*	*17%*	***56%***	*54%*	*58%*	***17%***	*16%*	*19%*	***2%***	*2%*	*3%*	***8%***	*7%*	*9%*				***1%***	*1%*	*2%*
Humber, Coast and Vale	**1,366**	***12%***	*10%*	*14%*	***66%***	*64%*	*69%*	***11%***	*10%*	*13%*	***3%***	*2%*	*4%*	***6%***	*5%*	*7%*				***2%***	*1%*	*3%*
Kent and Medway	**2,086**	***19%***	*17%*	*20%*	***43%***	*41%*	*45%*	***20%***	*18%*	*22%*	***10%***	*8%*	*11%*	***6%***	*5%*	*7%*				***3%***	*2%*	*4%*
Lancashire and South Cumbria	**1,938**	***20%***	*18%*	*22%*	***53%***	*51%*	*56%*	***14%***	*13%*	*16%*	***4%***	*3%*	*4%*	***7%***	*6%*	*9%*				***2%***	*1%*	*2%*
North Central and North East London	**2,488**	***24%***	*23%*	*26%*	***44%***	*42%*	*46%*	***15%***	*13%*	*16%*	***4%***	*3%*	*5%*	***10%***	*9%*	*11%*				***4%***	*3%*	*5%*
North East and Cumbria	**3,066**	***14%***	*13%*	*15%*	***62%***	*60%*	*64%*	***15%***	*14%*	*16%*	***3%***	*3%*	*4%*	***5%***	*4%*	*6%*				***1%***	*1%*	*2%*
North West and South West London	**3,028**	***16%***	*15%*	*17%*	***52%***	*51%*	*54%*	***16%***	*15%*	*18%*	***1%***	*1%*	*2%*	***10%***	*9%*	*11%*				***4%***	*4%*	*5%*
Peninsula	**1,680**	***12%***	*11%*	*14%*	***60%***	*57%*	*62%*	***20%***	*18%*	*22%*	***3%***	*2%*	*4%*	***4%***	*3%*	*5%*				***1%***	*1%*	*2%*
Somerset, Wiltshire, Avon and Gloucestershire	**2,571**	***17%***	*16%*	*19%*	***56%***	*54%*	*58%*	***19%***	*17%*	*20%*	***2%***	*1%*	*2%*	***5%***	*4%*	*6%*				***2%***	*2%*	*3%*
South East London	**1,382**	***14%***	*13%*	*16%*	***55%***	*53%*	*58%*	***15%***	*14%*	*17%*	***2%***	*1%*	*3%*	***8%***	*7%*	*10%*				***4%***	*4%*	*6%*
South Yorkshire, Bassetlaw, North Derbyshire and Hardwick	**1,636**	***18%***	*16%*	*20%*	***60%***	*57%*	*62%*	***14%***	*13%*	*16%*	***1%***	*1%*	*2%*	***6%***	*5%*	*8%*				***0%***	*0%*	*1%*
Surrey and Sussex	**4,716**	***17%***	*16%*	*19%*	***56%***	*55%*	*58%*	***13%***	*12%*	*14%*	***3%***	*3%*	*4%*	***6%***	*5%*	*7%*	***0%***	*0%*		***4%***	*3%*	*4%*
Thames Valley	**1,716**	***24%***	*22%*	*26%*	***41%***	*39%*	*43%*	***23%***	*21%*	*25%*	***4%***	*3%*	*5%*	***4%***	*3%*	*5%*				***5%***	*4%*	*6%*
Wessex	**2,304**	***27%***	*25%*	*29%*	***36%***	*34%*	*38%*	***22%***	*20%*	*23%*	***9%***	*8%*	*10%*	***5%***	*4%*	*6%*				***2%***	*2%*	*3%*
West Midlands	**4,561**	***23%***	*22%*	*24%*	***52%***	*50%*	*53%*	***15%***	*14%*	*16%*	***1%***	*1%*	*2%*	***6%***	*5%*	*7%*				***2%***	*2%*	*3%*
West Yorkshire and Harrogate	**2,074**	***15%***	*13%*	*17%*	***60%***	*58%*	*62%*	***12%***	*11%*	*14%*	***2%***	*2%*	*3%*	***8%***	*7%*	*10%*				***2%***	*2%*	*3%*
England	**50,571**	***19%***	*19%*	*20%*	***53%***	*52%*	*53%*	***16%***	*16%*	*17%*	***3%***	*3%*	*3%*	***6%***	*6%*	*7%*	***0%***	*0%*	*0%*	***3%***	*2%*	*3%*

MIBC	Number of MIBC cases	Two Week Wait	95% CI		GP referral	95% CI		Other Outpatient	95% CI		Inpatient Elective	95% CI		Emergency presentation	95% CI		Death Certificate Only	95% CI		Unknown	95% CI	
Cheshire and Merseyside	**5,084**	***32%***	*31%*	*34%*	***30%***	*29%*	*31%*	***15%***	*14%*	*16%*	***2%***	*2%*	*3%*	***17%***	*16%*	18%	**0%**	0%	1%	**2%**	2%	3%
East Midlands	**7,400**	***40%***	*39%*	*41%*	***24%***	*23%*	*25%*	***15%***	*15%*	*16%*	***2%***	*2%*	*3%*	***17%***	*16%*	18%	**0%**	0%	0%	**2%**	2%	2%
East of England	**11,429**	***36%***	*35%*	*37%*	***29%***	*29%*	*30%*	***11%***	*11%*	*12%*	***3%***	*2%*	*3%*	***17%***	*17%*	18%	**0%**	0%	0%	**3%**	3%	4%
Greater Manchester	**4,932**	***30%***	*29%*	*31%*	***33%***	*31%*	*34%*	***13%***	*13%*	*14%*	***2%***	*2%*	*2%*	***20%***	*19%*	22%	**0%**	0%	0%	**2%**	1%	2%
Humber, Coast and Vale	**3,054**	***30%***	*29%*	*32%*	***40%***	*38%*	*41%*	***7%***	*7%*	*8%*	***5%***	*4%*	*6%*	***16%***	*14%*	17%	**0%**	0%	0%	**2%**	2%	3%
Kent and Medway	**3,653**	***36%***	*35%*	*38%*	***24%***	*23%*	*26%*	***14%***	*13%*	*15%*	***3%***	*3%*	*4%*	***19%***	*17%*	20%	**1%**	1%	1%	**3%**	2%	4%
Lancashire and South Cumbria	**3,812**	***40%***	*38%*	*41%*	***25%***	*24%*	*26%*	***13%***	*12%*	*14%*	***4%***	*3%*	*4%*	***18%***	*16%*	19%	**0%**	0%	0%	**2%**	1%	2%
North Central and North East London	**3,540**	***27%***	*25%*	*28%*	***30%***	*28%*	*31%*	***12%***	*11%*	*13%*	***3%***	*2%*	*3%*	***24%***	*23%*	26%	**1%**	0%	1%	**4%**	3%	4%
North East and Cumbria	**6,255**	***42%***	*41%*	*43%*	***25%***	*24%*	*26%*	***12%***	*11%*	*13%*	***3%***	*3%*	*3%*	***17%***	*16%*	18%	**0%**	0%	0%	**1%**	1%	2%
North West and South West London	**4,141**	***29%***	*27%*	*30%*	***31%***	*30%*	*33%*	***12%***	*11%*	*13%*	***2%***	*1%*	*2%*	***21%***	*20%*	23%	**1%**	1%	1%	**5%**	4%	6%
Peninsula	**3,668**	***41%***	*40%*	*43%*	***24%***	*23%*	*26%*	***14%***	*13%*	*15%*	***3%***	*2%*	*3%*	***16%***	*15%*	17%	**0%**	0%	1%	**2%**	1%	2%
Somerset, Wiltshire, Avon and Gloucestershire	**5,294**	***39%***	*37%*	*40%*	***26%***	*25%*	*28%*	***12%***	*12%*	*13%*	***2%***	*2%*	*3%*	***17%***	*16%*	18%	**0%**	0%	1%	**3%**	3%	3%
South East London	**2,199**	***29%***	*27%*	*31%*	***33%***	*31%*	*35%*	***11%***	*10%*	*13%*	***1%***	*1%*	*2%*	***22%***	*20%*	24%	**0%**	0%	1%	**3%**	2%	3%
South Yorkshire, Bassetlaw, North Derbyshire and Hardwick	**3,448**	***38%***	*37%*	*40%*	***24%***	*23%*	*26%*	***16%***	*15%*	*17%*	***2%***	*2%*	*3%*	***18%***	*17%*	20%	**0%**	0%	1%	**1%**	1%	2%
Surrey and Sussex	**5,628**	***35%***	*34%*	*36%*	***30%***	*29%*	*31%*	***10%***	*10%*	*11%*	***3%***	*2%*	*3%*	***18%***	*17%*	19%	**0%**	0%	1%	**4%**	4%	5%
Thames Valley	**3,572**	***41%***	*40%*	*43%*	***22%***	*20%*	*23%*	***13%***	*12%*	*14%*	***5%***	*5%*	*6%*	***15%***	*14%*	16%	**0%**	0%	1%	**4%**	3%	5%
Wessex	**5,063**	***36%***	*34%*	*37%*	***24%***	*23%*	*25%*	***14%***	*13%*	*15%*	***6%***	*5%*	*7%*	***17%***	*16%*	19%	**0%**	0%	1%	**3%**	2%	3%
West Midlands	**9,892**	***40%***	*39%*	*41%*	***27%***	*27%*	*28%*	***11%***	*10%*	*11%*	***1%***	*1%*	*2%*	***18%***	*18%*	19%	**0%**	0%	0%	**2%**	2%	3%
West Yorkshire and Harrogate	**4,462**	***37%***	*36%*	*39%*	***26%***	*24%*	*27%*	***9%***	*8%*	*10%*	***4%***	*3%*	*4%*	***21%***	*20%*	23%	**0%**	0%	0%	**3%**	3%	4%
England	**96,526**	***36%***	*36%*	*37%*	***28%***	*27%*	*28%*	***12%***	*12%*	*13%*	***3%***	*3%*	*3%*	***18%***	*18%*	18%	**0%**	0%	0%	**3%**	3%	3%

Supplementary table 1. Routes to diagnosis for NMIBC and MIBC for each cancer alliance (from https://www.cancerdata.nhs.uk/routestodiagnosis/index).

Year	Tumour Type	Tumour Type 3	Stage	Stage Detail	Incidence (case number)	Chemotherapy (CT) only percentage	CT only percentage LCI	CT only percentage UCI	Radiotherapy (RT) only percentage	RT only percentage LCI	RT only percentage UCI	Surgery only percentage	Surgery only percentage LCI	Surgery only percentage UCI	CT and RT percentage	CT and RT percentage LCI	CT and RT percentage UCI	Surgery and CT percentage	Surgery and CT percentage LCI	Surgery and CT percentage UCI	Surgery and RT percentage	Surgery and RT percentage LCI	Surgery and RT percentage UCI	Surgery and CT and RT percentage	Surgery and CT and RT percentage LCI	Surgery and CT and RT percentage UCI	Other care percentage	Other care percentage LCI	Other care percentage UCI
2013	Renal Pelvis and Ureter	All	Non-muscle-invasive	T1	277	3.6%	2.0%	6.5%	2.2%	1.0%	4.6%	66.8%	61.0%	72.1%	0.4%	0.1%	2.0%	5.4%	3.3%	8.7%	1.8%	0.8%	4.2%	0.0%	0.0%	1.4%	19.9%	15.6%	25.0%
2013	Renal Pelvis and Ureter	All	Muscle-invasive	All	644	2.5%	1.5%	4.0%	1.1%	0.5%	2.2%	58.2%	54.4%	62.0%	0.9%	0.4%	2.0%	15.4%	12.8%	18.4%	4.2%	2.9%	6.0%	2.8%	1.8%	4.4%	14.9%	12.4%	17.9%
2013	Renal Pelvis and Ureter	All	Stage unknown	All	485	9.9%	7.5%	12.9%	8.7%	6.5%	11.5%	11.1%	8.6%	14.2%	2.5%	1.4%	4.3%	2.7%	1.6%	4.5%	0.6%	0.2%	1.8%	0.2%	0.0%	1.2%	64.3%	60.0%	68.5%
2013	Bladder	Urothelial	Non-muscle-invasive	T1	3599	1.3%	1.0%	1.7%	0.6%	0.4%	0.9%	50.9%	49.3%	52.5%	0.1%	0.1%	0.3%	39.5%	37.9%	41.1%	2.8%	2.3%	3.4%	1.9%	1.5%	2.4%	2.9%	2.4%	3.5%
2013	Bladder	Urothelial	Muscle-invasive	All	3290	7.6%	6.8%	8.6%	22.2%	20.8%	23.7%	11.9%	10.8%	13.0%	16.3%	15.1%	17.6%	11.6%	10.6%	12.8%	0.9%	0.6%	1.3%	1.2%	0.9%	1.7%	28.2%	26.7%	29.8%
2013	Bladder	Urothelial	Stage unknown	All	1490	8.7%	7.3%	10.2%	6.8%	5.7%	8.2%	1.1%	0.7%	1.8%	2.6%	1.9%	3.5%	1.3%	0.9%	2.1%	0.1%	0.0%	0.4%	0.1%	0.0%	0.5%	79.3%	77.1%	81.2%
2013	Bladder	Other	All stages	All	636	7.2%	5.5%	9.5%	13.2%	10.8%	16.1%	22.6%	19.6%	26.1%	10.7%	8.5%	13.3%	7.4%	5.6%	9.7%	2.4%	1.4%	3.9%	2.2%	1.3%	3.7%	34.3%	30.7%	38.1%
2013	Bladder	Other	Muscle-invasive	All	414	8.0%	5.7%	11.0%	14.7%	11.6%	18.5%	23.4%	19.6%	27.7%	14.0%	11.0%	17.7%	7.5%	5.3%	10.4%	2.2%	1.1%	4.1%	1.2%	0.5%	2.8%	29.0%	24.8%	33.5%
2013	Bladder	Other	Stage unknown	All	134	9.0%	5.2%	15.0%	17.2%	11.7%	24.4%	7.5%	4.1%	13.2%	7.5%	4.1%	13.2%	2.2%	0.8%	6.4%	0.0%	0.0%	2.8%	0.7%	0.1%	4.1%	56.0%	47.5%	64.1%
2014	Renal Pelvis and Ureter	All	Non-muscle-invasive	T1	299	3.0%	1.6%	5.6%	0.0%	0.0%	1.3%	64.5%	59.0%	69.8%	0.7%	0.2%	2.4%	7.4%	4.9%	10.9%	0.7%	0.2%	2.4%	0.0%	0.0%	1.3%	23.7%	19.3%	28.9%
2014	Renal Pelvis and Ureter	All	Muscle-invasive	All	673	2.5%	1.6%	4.0%	1.5%	0.8%	2.7%	56.6%	52.8%	60.3%	2.1%	1.2%	3.5%	17.1%	14.4%	20.1%	3.3%	2.2%	4.9%	2.5%	1.6%	4.0%	14.4%	12.0%	17.3%
2014	Renal Pelvis and Ureter	All	Stage unknown	All	443	7.9%	5.7%	10.8%	8.4%	6.1%	11.3%	9.5%	7.1%	12.6%	4.7%	3.1%	7.1%	0.9%	0.4%	2.3%	0.2%	0.0%	1.3%	0.2%	0.0%	1.3%	68.2%	63.7%	72.3%
2014	Bladder	Urothelial	Non-muscle-invasive	T1	3667	1.0%	0.8%	1.4%	0.3%	0.2%	0.6%	52.0%	50.3%	53.6%	0.1%	0.0%	0.2%	37.9%	36.3%	39.5%	2.8%	2.3%	3.4%	1.7%	1.3%	2.2%	4.2%	3.6%	4.9%
2014	Bladder	Urothelial	Muscle-invasive	All	3264	7.1%	6.3%	8.1%	25.4%	24.0%	27.0%	11.2%	10.1%	12.3%	15.6%	14.4%	16.8%	11.9%	10.8%	13.0%	0.8%	0.5%	1.2%	1.0%	0.7%	1.4%	27.0%	25.5%	28.6%
2014	Bladder	Urothelial	Stage unknown	All	1186	7.2%	5.8%	8.8%	5.9%	4.7%	7.4%	0.8%	0.4%	1.4%	2.2%	1.5%	3.2%	0.8%	0.4%	1.4%	0.0%	0.0%	0.3%	0.2%	0.0%	0.6%	83.1%	80.8%	85.1%
2014	Bladder	Other	All stages	All	617	6.2%	4.5%	8.3%	11.8%	9.5%	14.6%	26.1%	22.8%	29.7%	12.0%	9.7%	14.8%	6.5%	4.8%	8.7%	3.6%	2.4%	5.3%	2.4%	1.5%	4.0%	31.4%	27.9%	35.2%
2014	Bladder	Other	Muscle-invasive	All	391	5.4%	3.5%	8.1%	14.8%	11.7%	18.7%	23.5%	19.6%	28.0%	15.3%	12.1%	19.3%	7.2%	5.0%	10.2%	3.3%	2.0%	5.6%	1.3%	0.5%	3.0%	29.2%	24.9%	33.8%
2014	Bladder	Other	Stage unknown	All	122	10.7%	6.3%	17.4%	10.7%	6.3%	17.4%	16.4%	10.9%	24.0%	10.7%	6.3%	17.4%	2.5%	0.8%	7.0%	0.8%	0.1%	4.5%	0.8%	0.1%	4.5%	47.5%	38.9%	56.3%
2015	Renal Pelvis and Ureter	All	Non-muscle-invasive	T1	323	2.5%	1.3%	4.8%	0.9%	0.3%	2.7%	65.6%	60.3%	70.6%	0.3%	0.1%	1.7%	7.7%	5.3%	11.2%	0.6%	0.2%	2.2%	0.0%	0.0%	1.2%	22.3%	18.1%	27.1%
2015	Renal Pelvis and Ureter	All	Muscle-invasive	All	741	3.9%	2.7%	5.6%	1.9%	1.1%	3.1%	54.1%	50.5%	57.7%	1.6%	0.9%	2.8%	16.1%	13.6%	18.9%	3.2%	2.2%	4.8%	3.5%	2.4%	5.1%	15.7%	13.2%	18.4%
2015	Renal Pelvis and Ureter	All	Stage unknown	All	439	8.9%	6.6%	11.9%	7.5%	5.4%	10.4%	7.1%	5.0%	9.8%	4.3%	2.8%	6.7%	0.7%	0.2%	2.0%	0.5%	0.1%	1.6%	0.2%	0.0%	1.3%	70.8%	66.4%	74.9%
2015	Bladder	Urothelial	Non-muscle-invasive	T1	3615	0.9%	0.7%	1.3%	0.2%	0.1%	0.5%	52.0%	50.3%	53.6%	0.1%	0.1%	0.3%	38.8%	37.2%	40.4%	2.8%	2.3%	3.4%	1.9%	1.5%	2.4%	3.2%	2.7%	3.8%
2015	Bladder	Urothelial	Muscle-invasive	All	3313	7.4%	6.6%	8.3%	25.7%	24.2%	27.2%	10.2%	9.2%	11.3%	16.1%	14.9%	17.4%	12.3%	11.2%	13.4%	0.5%	0.3%	0.8%	0.8%	0.5%	1.1%	27.0%	25.5%	28.6%
2015	Bladder	Urothelial	Stage unknown	All	1162	5.9%	4.6%	7.4%	6.8%	5.5%	8.4%	0.3%	0.1%	0.9%	2.2%	1.5%	3.2%	0.3%	0.1%	0.9%	0.0%	0.0%	0.3%	0.2%	0.0%	0.6%	84.3%	82.1%	86.3%
2015	Bladder	Other	All stages	All	618	6.8%	5.1%	9.1%	14.7%	12.1%	17.7%	21.7%	18.6%	25.1%	9.7%	7.6%	12.3%	5.7%	4.1%	7.8%	2.9%	1.9%	4.6%	1.3%	0.7%	2.5%	37.2%	33.5%	41.1%
2015	Bladder	Other	Muscle-invasive	All	421	7.6%	5.4%	10.5%	18.8%	15.3%	22.8%	20.4%	16.9%	24.5%	12.4%	9.5%	15.8%	4.3%	2.7%	6.7%	1.9%	1.0%	3.7%	1.4%	0.7%	3.1%	33.3%	28.9%	37.9%
2015	Bladder	Other	Stage unknown	All	113	8.0%	4.2%	14.4%	8.9%	4.9%	15.5%	8.0%	4.2%	14.4%	6.2%	3.0%	12.2%	4.4%	1.9%	9.9%	0.0%	0.0%	3.3%	0.9%	0.2%	4.8%	63.7%	54.5%	72.0%
2016	Renal Pelvis and Ureter	All	Non-muscle-invasive	T1	307	2.9%	1.6%	5.5%	1.3%	0.5%	3.3%	63.8%	58.3%	69.0%	1.3%	0.5%	3.3%	7.2%	4.8%	10.6%	0.3%	0.1%	1.8%	0.0%	0.0%	1.2%	23.1%	18.8%	28.2%
2016	Renal Pelvis and Ureter	All	Muscle-invasive	All	708	4.4%	3.1%	6.1%	1.7%	1.0%	2.9%	54.9%	51.3%	58.6%	1.6%	0.9%	2.8%	20.2%	17.4%	23.3%	3.1%	2.1%	4.7%	1.8%	1.1%	3.1%	12.3%	10.1%	14.9%
2016	Renal Pelvis and Ureter	All	Stage unknown	All	484	6.0%	4.2%	8.5%	7.2%	5.2%	9.9%	7.9%	5.8%	10.6%	2.9%	1.7%	4.8%	1.0%	0.4%	2.4%	0.8%	0.3%	2.1%	0.0%	0.0%	0.8%	74.2%	70.1%	77.9%
2016	Bladder	Urothelial	Non-muscle-invasive	T1	3626	0.9%	0.7%	1.3%	0.2%	0.1%	0.4%	50.6%	49.0%	52.3%	0.1%	0.0%	0.3%	40.2%	38.6%	41.8%	3.0%	2.4%	3.6%	2.0%	1.6%	2.5%	3.1%	2.5%	3.7%
2016	Bladder	Urothelial	Muscle-invasive	All	3333	7.6%	6.7%	8.5%	25.1%	23.7%	26.6%	9.7%	8.7%	10.7%	16.3%	15.1%	17.6%	11.0%	10.0%	12.1%	0.8%	0.6%	1.2%	0.7%	0.4%	1.0%	28.9%	27.4%	30.4%
2016	Bladder	Urothelial	Stage unknown	All	1056	6.4%	5.1%	8.1%	5.8%	4.5%	7.4%	1.0%	0.6%	1.9%	1.6%	1.0%	2.6%	0.9%	0.4%	1.6%	0.1%	0.0%	0.5%	0.1%	0.0%	0.5%	84.1%	81.8%	86.2%
2016	Bladder	Other	All stages	All	610	6.2%	4.6%	8.4%	12.0%	9.6%	14.8%	28.9%	25.4%	32.6%	9.2%	7.1%	11.7%	6.6%	4.9%	8.8%	3.6%	2.4%	5.4%	1.5%	0.8%	2.8%	32.1%	28.5%	35.9%
2016	Bladder	Other	Muscle-invasive	All	398	6.8%	4.7%	9.7%	14.8%	11.7%	18.7%	25.4%	21.4%	29.9%	13.1%	10.1%	16.7%	7.0%	4.9%	10.0%	1.5%	0.7%	3.3%	1.0%	0.4%	2.6%	30.4%	26.1%	35.1%
2016	Bladder	Other	Stage unknown	All	109	7.3%	3.8%	13.8%	11.9%	7.1%	19.3%	17.4%	11.5%	25.6%	3.7%	1.4%	9.1%	0.9%	0.2%	5.0%	0.9%	0.2%	5.0%	0.9%	0.2%	5.0%	56.9%	47.5%	65.8%
2017	Renal Pelvis and Ureter	All	Non-muscle-invasive	T1	256	2.3%	1.1%	5.0%	1.6%	0.6%	3.9%	65.2%	59.2%	70.8%	0.8%	0.2%	2.8%	8.2%	5.4%	12.2%	0.4%	0.1%	2.2%	0.4%	0.1%	2.2%	21.1%	16.5%	26.5%
2017	Renal Pelvis and Ureter	All	Muscle-invasive	All	717	3.8%	2.6%	5.4%	1.7%	1.0%	2.9%	51.9%	48.2%	55.5%	1.4%	0.8%	2.5%	21.2%	18.4%	24.3%	2.2%	1.4%	3.6%	2.9%	1.9%	4.4%	14.9%	12.5%	17.7%
2017	Renal Pelvis and Ureter	All	Stage unknown	All	464	11.4%	8.8%	14.6%	6.3%	4.4%	8.8%	4.1%	2.6%	6.3%	4.3%	2.8%	6.6%	1.3%	0.6%	2.8%	0.0%	0.0%	0.8%	0.2%	0.0%	1.2%	72.4%	68.2%	76.3%
2017	Bladder	Urothelial	Non-muscle-invasive	T1	3579	1.1%	0.8%	1.5%	0.2%	0.1%	0.4%	43.5%	41.9%	45.2%	0.1%	0.0%	0.2%	48.2%	46.6%	49.9%	2.3%	1.9%	2.9%	2.0%	1.6%	2.5%	2.5%	2.1%	3.1%
2017	Bladder	Urothelial	Muscle-invasive	All	3508	10.6%	9.7%	11.7%	21.0%	19.7%	22.4%	9.4%	8.5%	10.4%	16.8%	15.6%	18.0%	11.5%	10.5%	12.6%	0.5%	0.3%	0.8%	0.7%	0.4%	1.0%	29.5%	28.0%	31.0%
2017	Bladder	Urothelial	Stage unknown	All	1091	8.2%	6.8%	10.0%	5.0%	3.8%	6.4%	0.6%	0.3%	1.2%	1.7%	1.0%	2.6%	0.6%	0.3%	1.3%	0.1%	0.0%	0.5%	0.0%	0.0%	0.4%	83.9%	81.6%	85.9%
2017	Bladder	Other	All stages	All	646	7.1%	5.4%	9.4%	10.7%	8.5%	13.3%	23.2%	20.1%	26.6%	10.8%	8.7%	13.5%	9.0%	7.0%	11.4%	2.0%	1.2%	3.4%	1.2%	0.6%	2.4%	35.9%	32.3%	39.7%
2017	Bladder	Other	Muscle-invasive	All	445	7.9%	5.7%	10.7%	13.5%	10.6%	17.0%	19.3%	15.9%	23.3%	13.9%	11.0%	17.5%	7.6%	5.5%	10.5%	2.0%	1.1%	3.8%	0.7%	0.2%	2.0%	35.1%	30.8%	39.6%
2017	Bladder	Other	Stage unknown	All	108	9.3%	5.1%	16.2%	8.3%	4.4%	15.1%	13.0%	7.9%	20.6%	7.4%	3.8%	13.9%	7.4%	3.8%	13.9%	0.0%	0.0%	3.4%	0.9%	0.2%	5.1%	53.7%	44.3%	62.8%
2018	Renal Pelvis and Ureter	All	Non-muscle-invasive	T1	261	7.7%	5.0%	11.5%	0.8%	0.2%	2.8%	57.1%	51.0%	62.9%	0.4%	0.1%	2.1%	9.6%	6.6%	13.8%	0.4%	0.1%	2.1%	0.0%	0.0%	1.5%	24.1%	19.3%	29.7%
2018	Renal Pelvis and Ureter	All	Muscle-invasive	All	704	5.4%	4.0%	7.3%	0.7%	0.3%	1.7%	43.0%	39.4%	46.7%	1.3%	0.7%	2.4%	30.5%	27.3%	34.0%	0.6%	0.2%	1.5%	2.3%	1.4%	3.7%	16.2%	13.7%	19.1%
2018	Renal Pelvis and Ureter	All	Stage unknown	All	488	11.5%	8.9%	14.6%	8.8%	6.6%	11.7%	5.5%	3.8%	7.9%	3.9%	2.5%	6.0%	2.9%	1.7%	4.8%	0.4%	0.1%	1.5%	0.2%	0.0%	1.2%	66.8%	62.5%	70.8%
2018	Bladder	Urothelial	Non-muscle-invasive	T1	3664	0.8%	0.5%	1.1%	0.1%	0.1%	0.3%	44.6%	43.0%	46.2%	0.1%	0.0%	0.3%	47.8%	46.2%	49.4%	2.3%	1.8%	2.8%	1.7%	1.3%	2.1%	2.6%	2.2%	3.2%
2018	Bladder	Urothelial	Muscle-invasive	All	3396	10.7%	9.7%	11.8%	20.6%	19.3%	22.0%	9.8%	8.9%	10.9%	15.8%	14.6%	17.0%	13.6%	12.5%	14.8%	0.5%	0.3%	0.8%	0.8%	0.5%	1.2%	28.3%	26.8%	29.8%
2018	Bladder	Urothelial	Stage unknown	All	1083	7.8%	6.4%	9.6%	5.5%	4.3%	7.1%	0.3%	0.1%	0.8%	2.7%	1.9%	3.8%	0.5%	0.2%	1.1%	0.0%	0.0%	0.4%	0.0%	0.0%	0.4%	83.2%	80.9%	85.3%
2018	Bladder	Other	All stages	All	629	9.7%	7.6%	12.3%	10.8%	8.6%	13.5%	22.7%	19.6%	26.2%	11.3%	9.0%	14.0%	7.8%	5.9%	10.2%	2.1%	1.2%	3.5%	3.2%	2.1%	4.9%	32.4%	28.9%	36.2%
2018	Bladder	Other	Muscle-invasive	All	417	8.9%	6.5%	12.0%	13.7%	10.7%	17.3%	21.1%	17.5%	25.3%	13.7%	10.7%	17.3%	6.7%	4.7%	9.5%	1.4%	0.7%	3.1%	2.6%	1.5%	4.7%	31.9%	27.6%	36.5%
2018	Bladder	Other	Stage unknown	All	102	17.6%	11.5%	26.2%	9.8%	5.4%	17.1%	7.8%	4.0%	14.7%	13.7%	8.4%	21.7%	2.0%	0.5%	6.9%	0.0%	0.0%	3.6%	0.0%	0.0%	3.6%	49.0%	39.5%	58.6%
2019	Renal Pelvis and Ureter	All	Non-muscle-invasive	T1	292	3.8%	2.1%	6.6%	1.0%	0.4%	3.0%	63.7%	58.0%	69.0%	2.1%	0.9%	4.4%	9.9%	7.0%	13.9%	0.3%	0.1%	1.9%	0.0%	0.0%	1.3%	19.2%	15.1%	24.1%
2019	Renal Pelvis and Ureter	All	Muscle-invasive	All	638	5.3%	3.8%	7.4%	1.9%	1.1%	3.3%	40.4%	36.7%	44.3%	1.7%	1.0%	3.1%	31.7%	28.2%	35.4%	2.7%	1.7%	4.2%	2.2%	1.3%	3.6%	14.1%	11.6%	17.0%
2019	Renal Pelvis and Ureter	All	Stage unknown	All	472	10.0%	7.6%	13.0%	6.1%	4.3%	8.7%	7.8%	5.7%	10.6%	2.8%	1.6%	4.7%	2.1%	1.2%	3.9%	0.2%	0.0%	1.2%	0.0%	0.0%	0.8%	71.0%	66.7%	74.9%
2019	Bladder	Urothelial	Non-muscle-invasive	T1	3518	1.0%	0.7%	1.4%	0.2%	0.1%	0.4%	47.2%	45.6%	48.9%	0.0%	0.0%	0.2%	44.5%	42.8%	46.1%	1.8%	1.5%	2.3%	2.4%	2.0%	3.0%	2.8%	2.3%	3.4%
2019	Bladder	Urothelial	Muscle-invasive	All	3269	9.9%	8.9%	11.0%	20.6%	19.2%	22.0%	10.6%	9.5%	11.7%	17.7%	16.4%	19.1%	11.0%	10.0%	12.1%	0.3%	0.2%	0.6%	0.7%	0.5%	1.1%	29.2%	27.7%	30.8%
2019	Bladder	Urothelial	Stage unknown	All	1481	8.9%	7.6%	10.5%	5.7%	4.7%	7.0%	0.9%	0.6%	1.6%	2.2%	1.6%	3.1%	1.6%	1.1%	2.4%	0.0%	0.0%	0.3%	0.1%	0.0%	0.4%	80.5%	78.4%	82.4%
2019	Bladder	Other	All stages	All	709	7.6%	5.9%	9.8%	7.8%	6.0%	10.0%	24.5%	21.5%	27.8%	9.9%	7.9%	12.3%	9.6%	7.6%	12.0%	2.8%	1.8%	4.3%	2.3%	1.4%	3.6%	35.5%	32.1%	39.1%
2019	Bladder	Other	Muscle-invasive	All	458	7.6%	5.5%	10.4%	9.0%	6.7%	11.9%	23.1%	19.5%	27.2%	13.8%	10.9%	17.2%	9.2%	6.9%	12.2%	1.5%	0.7%	3.1%	1.1%	0.5%	2.5%	34.7%	30.5%	39.2%
2019	Bladder	Other	Stage unknown	All	120	14.2%	9.0%	21.5%	9.2%	5.2%	15.7%	7.5%	4.0%	13.6%	5.8%	2.9%	11.6%	4.2%	1.8%	9.4%	0.0%	0.0%	3.1%	0.0%	0.0%	3.1%	59.2%	50.2%	67.5%

Supplementary table 2. Treatment choices for all cancers within this dataset.
